# Prefoldin and Pins synergistically regulate asymmetric division and suppress dedifferentiation

**DOI:** 10.1038/srep23735

**Published:** 2016-03-30

**Authors:** Yingjie Zhang, Madhulika Rai, Cheng Wang, Cayetano Gonzalez, Hongyan Wang

**Affiliations:** 1 Neuroscience & Behavioral Disorders Program, Duke-NUS Graduate Medical School Singapore, 8 College Road, Singapore 169857; 2 NUS Graduate School for Integrative Sciences and Engineering, National University of Singapore, 28 Medical Drive, Singapore 117456; 3 Institute for Research in Biomedicine (IRB-Barcelona), Baldiri Reixac 10, 08028 Barcelona, Spain; 4 Institució Catalana de Recerca i Estudis Avançats (ICREA). Passeig Lluís Companys 23, 08010 Barcelona, Spain; 5 Dept. of Physiology, Yong Loo Lin School of Medicine, National University of Singapore, Singapore 117597.

## Abstract

Prefoldin is a molecular chaperone complex that regulates tubulin function in mitosis. Here, we show that Prefoldin depletion results in disruption of neuroblast polarity, leading to neuroblast overgrowth in *Drosophila* larval brains. Interestingly, co-depletion of Prefoldin and Partner of Inscuteable (Pins) leads to the formation of gigantic brains with severe neuroblast overgrowth, despite that Pins depletion alone results in smaller brains with partially disrupted neuroblast polarity. We show that Prefoldin acts synergistically with Pins to regulate asymmetric division of both neuroblasts and Intermediate Neural Progenitors (INPs). Surprisingly, co-depletion of Prefoldin and Pins also induces dedifferentiation of INPs back into neuroblasts, while depletion either Prefoldin or Pins alone is insufficient to do so. Furthermore, knocking down either *α-tubulin* or *β-tubulin* in *pins*^*-*^ mutant background results in INP dedifferentiation back into neuroblasts, leading to the formation of ectopic neuroblasts. Overexpression of α-tubulin suppresses neuroblast overgrowth observed in *prefoldin pins* double mutant brains. Our data elucidate an unexpected function of Prefoldin and Pins in synergistically suppressing dedifferentiation of INPs back into neural stem cells.

Control of tissue homeostasis is a central issue during development. The neural stem cells, or neuroblasts, of the *Drosophila* larval brain is an excellent model for studying stem cell homeostasis^1^,^2^,^3^,^4^,^5^. Asymmetric division of neuroblasts generates a self-renewing neuroblast and a different daughter cell that undergoes differentiation pathway to produce neurons or glia^6^. Following each asymmetric division, apical proteins such as aPKC are segregated into the neuroblast daughter and function as “proliferation factor”, while basal proteins are segregated into a smaller daughter cell to act as “differentiation factors”^7^,^8^,^9^,^10^. At the onset of mitosis, the Partitioning defective (Par) protein complex that is composed of Bazooka (Baz)/Par3, Par6 and atypical protein kinase C (aPKC) is asymmetrically localized at the apical cortex of the neuroblast^11^,^12^,^13^. Other apical proteins including Partner of Inscuteable (Pins), the heterotrimeric G protein Gαi, and Mushroom body defect (Mud) also accumulate at the apical cortex through an interaction of Inscuteable (Insc) with Par protein complex^14^,^15^,^16^,^17^,^18^. Apical proteins control basal localization of cell fate determinants Numb, Prospero (Pros), Brain tumor (Brat) and their adaptor proteins Miranda (Mira) and Partner of Numb (Pon) that are segregated into the ganglion mother cell (GMC) following divisions^1^. Apical proteins and their regulators also control mitotic spindle orientation to ensure correct asymmetric protein segregation at telophase^14^,^15^,^16^,^17^,^18^,^19^,^20^,^21^,^22^,^23^. Several centrosomal proteins, Aurora A, Polo and Centrosomin, regulate mitotic spindle orientation^24^,^25^,^26^.

There are at least two different types of neuroblasts that undergo asymmetric division in the larval central brain^27^,^28^,^29^. Perturbation of asymmetric division in either type of neuroblast can trigger neuroblast overproliferation and/or the induction of brain tumors^4^,^30^. The majority of neuroblasts are type I neuroblasts that generate a neuroblast and a GMC in each division, while type II neuroblasts generate a neuroblast and an intermediate neural progenitor (INP), which undergoes three to five rounds of asymmetric division to produce GMCs^27^,^28^,^29^. Ets transcription factor Pointed (PntP1 isoform), exclusively expressed in type II neuroblast lineages, promotes the formation of INPs^31^. Failure to restrict the self-renewal potential of INPs can lead to dedifferentiation, allowing INPs to revert back into “ectopic neuroblasts”^32^. Notch antagonist Numb and Brat function cooperatively to promote the INP fate^29^. Loss of *brat* or *numb* leads to “ectopic type II neuroblasts” originating from uncommitted immature INPs that failed to undergo maturation^29^. A zinc-finger transcription factor Earmuff functions after Brat and Numb in immature INPs to prevent their dedifferentiation^33^. Earmuff also associates with Brahma and HDAC3, which are involved in chromatin remodeling, to prevent INP dedifferentiation^34^,^35^. However, the underlying mechanism by which INPs possess limited developmental potential is largely unknown.

Prefoldin (Pfdn) was first identified as a hetero-hexameric chaperone consisting of two α-like (PFDN3 and 5) and four β-like (PFDN 1, 2, 4 and 6) subunits, based on its ability to capture unfolded actin^36^. Prefoldin promotes folding of proteins such as tubulin and actin by binding specifically to cytosolic chaperonin containing TCP-1 (CCT) and by directing target proteins to it^36^. The yeast homologs of Prefoldin 2–6, named GIM1-5 (genes involved in microtubule biogenesis) are present in a complex that facilitates proper folding of α-tubulin and γ-tubulin^37^. All Prefoldin subunits are phylogenetically conserved from Archaea to Eukarya^38^. Structural study of the Prefoldin hexamer from the archaeum *M. thermoautotrophicum* showed that Prefoldin forms a jellyfish-like shape consisting of a double β barrel assembly with six long tentacle-like coiled coils that participate in substrate binding^39^. The function of Prefoldin as a chaperone has also been illustrated in lower eukaryotes like *C. elegans*, in which loss of *prefoldin* resulted in defects in cell division due to reduced microtubule growth rate^40^. Depletion of *PFDN1* in mice displayed cytoskeleton-related defects, including neuronal loss and lymphocyte development defects^41^. The only Prefoldin subunit in *Drosophila* that has been characterized to date, Merry-go-round (Mgr), the Pfdn3 subunit, cooperates with the tumor suppressor Von Hippel Lindau (VHL) to regulate tubulin stability^42^. However, the functions of Prefoldin in the nervous system remain elusive.

Here, we describe the critical role of evolutionarily-conserved Prefoldin complex in regulating neuroblast and INP asymmetric division and suppressing INP dedifferentiation. Mutants for two Prefoldin subunits, Mgr and Pfdn2, displayed neuroblast overgrowth with defects in cortical polarity of Par proteins and microtubule-related abnormalities. Interestingly, co-depletion of Pins in *mgr* or *pfdn2* mutants led to massive neuroblast overgrowth. Prefoldin and Pins synergistically regulate asymmetric division of both neuroblasts and INPs. Surprisingly, they also synergistically suppress dedifferentiation of INPs back into neuroblasts. Knocking down *tubulins* in *pins* mutant background resulted in severe neuroblasts overgrowth, mimicking that caused by co-depletion of Prefoldin and Pins. Our data provide a new mechanism by which Prefoldin and Pins regulates neural stem cell homeostasis through regulating tubulin stability in both neuroblasts and INPs.

## Results

### Pfdn2 depletion results in the formation of ectopic neuroblasts

**Figure 1:**
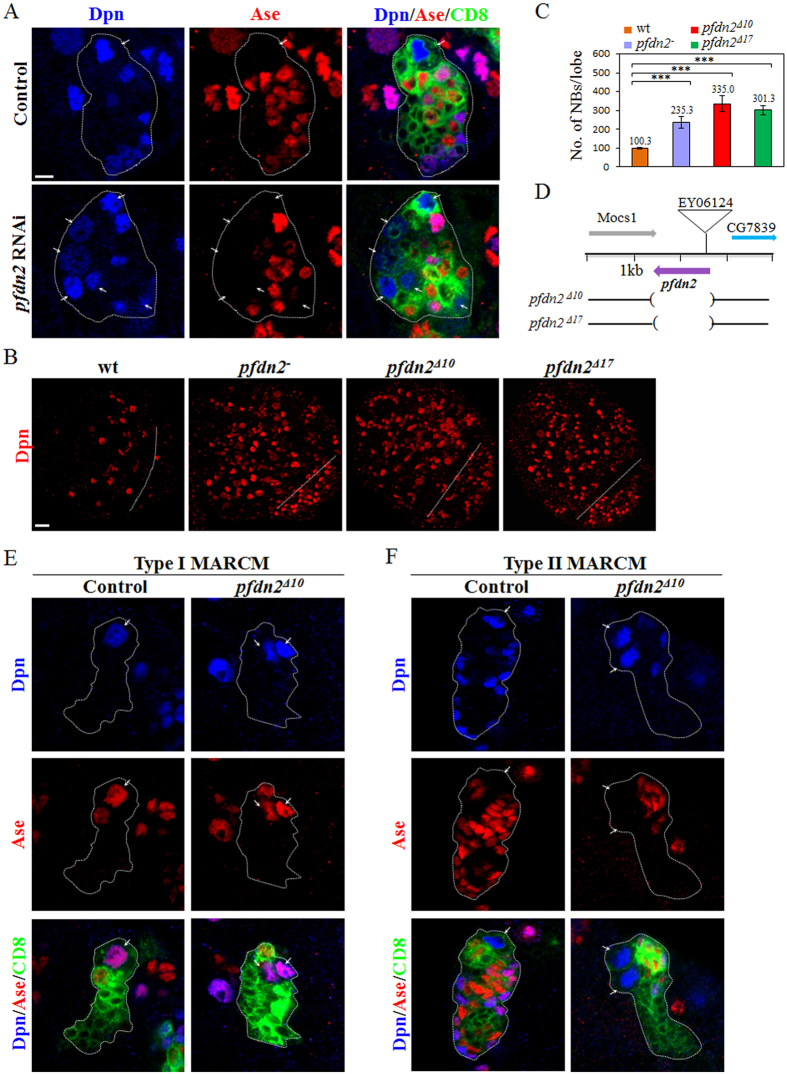
Pfdn2 suppresses neuroblast overproliferation in larval brains. (**A**) Type II driver control (*wor*-Gal4 *ase*-Gal80 UAS-CD8-GFP) and *pfdn2* RNAi were labeled with Dpn, Ase and CD8. (**B**) Dpn was labeled in wild-type, *pfdn2*^−^ [*pfdn2*^*01239*^/*Df(3L)BSC457*], *pfdn2*^Δ*10*^, and *pfdn2*^Δ*17*^ larval brains. The central brain (CB) is to the left of the white dotted line, which markers the border between the CB and the optic lobe (OL). (**C**) Quantification of larval brain neuroblasts. ***indicates p < 0.001. Error bars indicate mean standard deviation. NBs, neuroblasts. (**D**) Schematic representation of *pfdn2* genomic locus and the *pfdn2* deletion alleles used for this study. The extent of the deletion is indicated by the parentheses. (**E**,**F**) MARCM driver control and *pfdn2*^Δ*10*^ MARCM clones were labeled with Dpn, Ase and CD8. Neuroblasts (Dpn^+^ Ase^+^ in type I lineages and Dpn^+^ Ase^−^ in type II lineages) in the clones are indicated by arrows. Individual neuroblast clones or lineages are marked by CD8::GFP and outlined by white dotted lines. Scale bars: 5 μm (**A**,**E**–**F**), 20 μm (**B**).

We identified *pfdn2/CG6302*, encoding a Prefoldin β-like subunit, from a RNA interference (RNAi) screen in larval brains (Zhang Y and Wang H, unpublished data). Ectopic neuroblasts labeled by a neuroblast marker, Deadpan (Dpn), were formed upon knocking down *pfdn2* under a neuroblast driver *insc*-Gal4 (Fig. S1A). Only one neuroblast was observed in control type I neuroblast lineages using *insc*-Gal4 (Fig. S1B; 100%, n = 40) and type II neuroblast lineages using *worniu*-Gal4 with *asense (ase)*-Gal80^43^ (Fig. 1A; 100%, n = 40). In contrast, upon *pfdn2* RNAi excess neuroblasts were observed in both type I neuroblast lineages (Fig. S1B; 53.1%, n = 32) and type II neuroblast lineages (Fig. 1A; 75.0%, n = 32), respectively. To verify the function of Pfdn2 in neuroblasts, we analyzed a putative hypomorphic allele of *pfdn2*, *pfdn2*^*01239*^, which has a P element inserted at the 5′ untranslated region (UTR) of *pfdn2*. Hemizygous larval brains of *pfdn2*^*01239*^ over *Df(3L)BSC457* (referred to as *pfdn2*^*−*^ thereafter) displayed 235.3 ± 31.7 neuroblasts per brain hemisphere (Fig. 1B–C, n = 25), suggesting that Pfdn2 inhibits the formation of ectopic neuroblasts in larval brains. Consistently, an increase of EdU (5-ethynyl-2′-deoxyuridine)-incorporation was also observed in *pfdn2*^*−*^ mutants compared to the control (Fig. S1C). To generate *pfdn2* null alleles, we mobilized a P element, EY06124. Its imprecise excision yielded two loss-of-function alleles, *pfdn2*^Δ*10*^ and *pfdn2*^Δ*17*^, both deleting the entire opening reading frame (ORF) of *pfdn2* (Fig. 1D). *pfdn2*^Δ*10*^ and *pfdn2*^Δ*17*^ mutants survive to pupal stage and display strong phenotypes with ectopic neuroblasts labeled by Dpn (Fig. 1B–C; 335.0 ± 42.6 neuroblasts/lobe, n = 32 and 301.3 ± 22.7 neuroblasts/lobe, n = 25, respectively). These phenotypes in *pfdn2*^Δ*10*^ and *pfdn2*^Δ*17*^ mutant brains can be fully rescued by overexpression of wild-type *pfdn2* or *pfdn2-Venus* transgene (Fig. S1D–F). Pfdn2 is abundantly expressed in neuroblasts, INPs and their immediate neural progeny- GMCs, detected by a specific antibody generated against Pfdn2 full length (Fig. S1G) and a transgenic Pfdn2 with a Venus tag at the C-terminus (Fig. S1J). In addition, Pfdn2 expression under the *tubulin*-Gal4 fully rescued the lethality of both *pfdn2*^Δ*10*^ and *pfdn2*^Δ*17*^ mutants. Pfdn2 protein was undetectable in *pfdn2*^Δ*10*^ zygotic mutants (Fig. S1G–H), further supporting that it is a null allele. Both type I and type II MARCM (Mosaic Analysis with Repressible Cell Marker)^44^ clones of *pfdn2*^Δ*10*^ generated excess neuroblasts (Fig. 1E–F; type I, 41.2%, n = 34; type II, 25.0%, n = 20). These phenotypes were slightly weaker than *pfdn2*^Δ*10*^ zygotic mutants, likely due to residual Pfdn2 protein in the clones (Fig. S1I). These data indicate that Pfdn2 is required in both type I and type II neuroblast lineages to prevent the formation of ectopic neuroblasts.

### The Prefoldin complex suppresses the formation of ectopic neuroblasts

**Figure 2:**
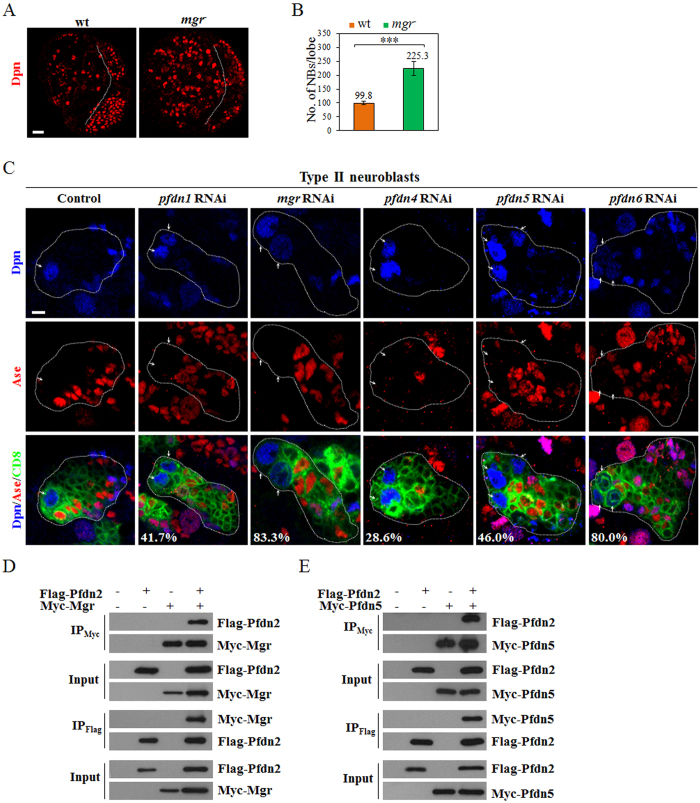
Prefoldin is important for neuroblast homeostasis. (**A**) Dpn was labeled in wild-type and *mgr*^*−*^ [*mgr*^*G5308*^/*Df(3R)Exel6160*] larval brains. The central brain (CB) is to the left of the white dotted line, which markers the border between the CB and the optic lobe. (**B**) Quantification of larval brain neuroblasts. ***indicates p < 0.001. Error bars indicate mean standard deviation. NBs, neuroblasts. (**C**) Type II driver control (*wor*-Gal4 *ase*-Gal80 UAS-CD8-GFP), *pfdn1* RNAi, *mgr* RNAi, *pfdn4* RNAi, *pfdn5* RNAi and *pfdn6* RNAi were labeled with Dpn, Ase and CD8. Neuroblasts (Dpn^+^ Ase^-^ in type II lineages) in the clones are indicated by arrows. Clones are marked by CD8::GFP and outlined by white dotted lines. (**D**,**E**) Co-IPs of S2 cells co-expressing Flag-Pfdn2, Myc-Mgr (**D**) or Flag-Pfdn2, Myc-Pfdn5 (**E**). Scale bars: 5 μm (**C**), 20 μm (**A**).

The full chaperone activity of the Prefoldin complex requires all six subunits^39^. Therefore, we ascertained the potential role of other Prefoldin subunits in neuroblasts. We generated a hemizygous *mgr*^*−*^ mutant with *mgr*^*G5308*^, a putative *mgr* mutant with a P element inserted at the 5′UTR of *mgr* gene, and a deficiency *Df(3R)Exel6160* that deletes the entire *mgr* gene. This *mgr*^*−*^ mutant accumulated ectopic neuroblasts in larval brains (Fig. 2A–B; 225.3 ± 25.0 neuroblasts/lobe, n = 35), suggesting that Mgr suppresses the formation of ectopic neuroblasts, similar to Pfdn2. Consistently, *mgr* RNAi knockdown led to ectopic neuroblasts in both type I (Fig. S2; 67.6%, n = 34) and type II neuroblast lineages (Fig. 2C; 83.3%, n = 30). Furthermore, RNAi knockdown of any of other four Prefoldin genes *pfdn1/CG13993*, *pfdn4/CG10635*, *pfdn5/CG7048* or *pfdn6/CG7770*, resulted in ectopic neuroblasts in both type I (Fig. S2; *pfdn1* RNAi, 43.2%, n = 37; *pfdn4* RNAi, 26.3%, n = 38; *pfdn5* RNAi, 38.2%, n = 34; *pfdn6* RNAi, 68.4%, n = 38) and type II neuroblast lineages (Fig. 2C; *pfdn1* RNAi, 41.7%, n = 36; *pfdn4* RNAi, 28.6%, n = 35; *pfdn5* RNAi, 46.0%, n = 50; *pfdn6* RNAi, 80.0%, n = 40). To ascertain that these Prefoldin subunits form a protein complex as predicted, we carried out co-immunoprecipitation experiments in S2 cells. Indeed, Flag-tagged Pfdn2 physically associates with Myc-tagged Mgr in co-immunoprecipitation experiments (Fig. 2D). Likewise, Myc-tagged Pfdn5 physically interacts with Flag-tagged Pfdn2 (Fig. 2E). These data indicate that the Prefoldin complex is important for inhibition of the formation of ectopic neuroblasts.

### Mgr and Pfdn2 regulate Par polarity in neuroblasts

**Figure 3:**
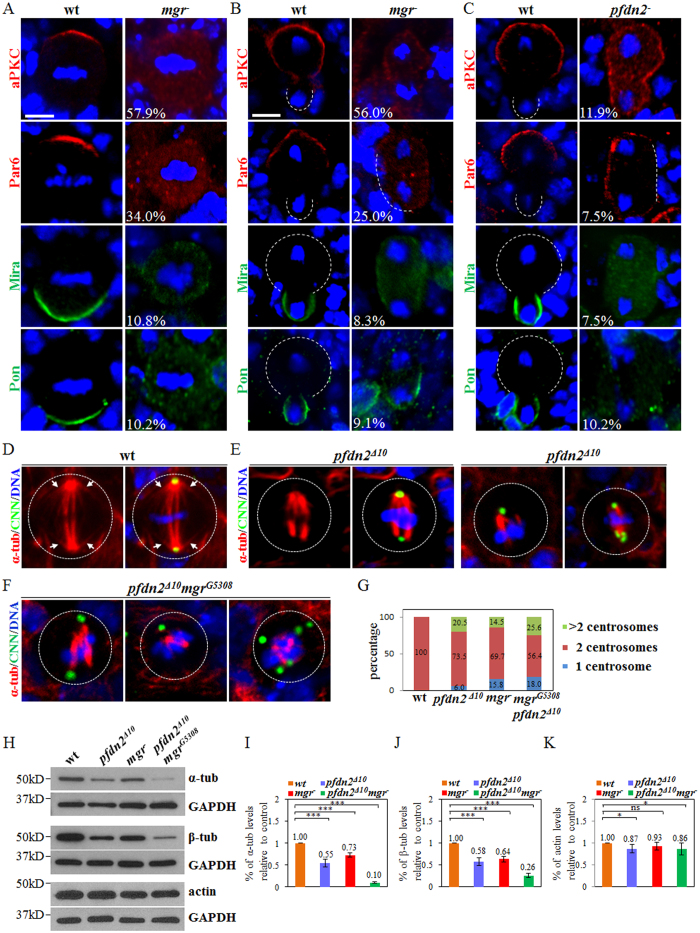
Pfdn2 and Mgr regulate neuroblast asymmetric division and mitotic spindle assembly. (**A**,**B**) Larval brains of wild-type and *mgr*^*−*^ [*mgr*^*G5308*^/*Df(3R)Exel6160*] hemizygote larvae were labeled with aPKC, Par6, Mira, Pon and DNA. (**C**) aPKC, Par6, Mira, Pon and DNA were labeled in wild-type and *pfdn2*^*−*^ [*pfdn2*^*01239*^/*Df(3L)BSC457*] hemizygote larvae. (D-F) Wild-type (**D**), *pfdn2*^Δ*10*^ (**E**) and *pfdn2*^Δ*10*^ *mgr*^*G5308*^ (**F**) larval brains were labeled with CNN, α-tubulin and DNA. Astral microtubules are indicated by white arrows and neuroblasts are outlined by white dotted lines. (**G**) Quantification of centrosome numbers. (**H**–**K**) Western blot of α-tubulin, β-tubulin and actin. Protein extracts from wild-type, *pfdn2*^Δ*10*^, *mgr*^*−*^ [*mgr*^*G5308*^/*Df(3R)Exel6160*] hemizygote and *pfdn2*^Δ*10*^ *mgr*^*G5308*^ larval brains were probed by anti-α-tubulin, anti-β-tubulin and anti-actin. GAPDH is loading control. The expression levels of α-tubulin (**I**), β-tubulin (**J**) and actin (**K**) were quantified. ***indicates p < 0.001, *indicates p < 0.05, ns indicates p > 0.05. Error bars indicate mean standard deviation. Scale bars: 5 μm (**A**,**D**–**F**), 4 μm (**B**–**C**).

We wondered if Prefoldin is required for asymmetric division of neuroblasts to prevent the formation of ectopic neuroblasts. Indeed, *mgr*^*−*^ mutant neuroblasts showed prominent defects of asymmetric division. In wild-type prometa/metaphase neuroblasts, aPKC and Par6 were always asymmetrically localized at the apical cortex while Mira and Pon were localized at the basal cortex (Fig. 3A, 100%, n = 50). Interestingly, aPKC was delocalized in 57.9% of the neuroblasts in *mgr* mutants during prometa/metaphase (Fig. 3A; n = 126). Similarly, Par6 was delocalized in 34.0% of prometa/metaphase in *mgr*^*−*^ neuroblasts (Fig. 3A; n = 106). Basal proteins Mira (Fig. 3A; 10.8%, n = 102) and Pon (Fig. 3A; 10.2%, n = 108) were mildly disrupted in *mgr*^*−*^ neuroblasts during prometa/metaphase. In contrast, neither Mgr nor Pfdn2 was important for Pins/Gαi cortical polarity (Fig. S3A–B). In most of previously reported mutants, asymmetric localization defects at prometa/metaphase were mostly restored during telophase by a poorly understood mechanism termed “telophase rescue”^45^. Surprisingly, all these proteins including aPKC (Fig. 3B; 56.0%, n = 25), Par6 (Fig. 3B; 25.0%, n = 32), Mira (Fig. 3B; 8.3%, n = 36) and Pon (Fig. 3B; 9.1%, n = 22) were still mis-segregated during telophase in *mgr* mutant neuroblasts, suggesting that telophase rescue did not happen in *mgr*^*−*^ mutants. Therefore, Mgr is crucial for cortical polarity of Par proteins and asymmetric protein segregation in neuroblasts.

*pfdn2* mutants also displayed defects of asymmetric protein localization of aPKC (Fig. S3C; *pfdn2*^Δ*10*^, 26.5%, n = 49; *pfdn2*^*−*^, 19.3%, n = 119) and Par6 (Fig. S3C; *pfdn2*^Δ*10*^, 15.2%, n = 46; *pfdn2*^*−*^, 13.7%, n = 95) during prometa/metaphase, albeit with weaker phenotypes. Interestingly, during telophase, mis-segregation of these proteins were evident in *pfdn2*^*−*^ mutants (Fig. 3C; aPKC, 11.9%, n = 109; Par6, 7.5%, n = 107; Mira, 7.5%, n = 107; Pon, 10.2%, n = 118), suggesting that Pfdn2 is also important for asymmetric protein segregation at telophase. The defects in asymmetric protein segregation observed in *pfdn2*^*−*^ and *mgr*^*−*^ mutants were likely responsible for the neuroblast overgrowth.

In wild-type metaphase neuroblasts, the mitotic spindle marked by α-tubulin is aligned with the apicobasal axis (inferred by apical Baz) (Fig. S3D; 100%, n = 50), which is important for asymmetric protein segregation at telophase. In both *mgr*^*−*^ (Fig. S3D; 23.7%, n = 76) and *pfdn2*^Δ*10*^ (Fig. S3D; 20.0%, n = 85) mutant neuroblasts, mitotic spindles were mis-oriented with apicobasal axis in cells that still retained asymmetric Baz localization. Taken together, both Mgr and Pfdn2 are required for Par polarity and mitotic spindle orientation in neuroblasts.

### Pfdn2 is critical for centrosomal functions

Prefoldin is important for the assembly of αβ-tubulin dimers^37^,^46^ and *mgr* mutant cells have microtubule-based abnormalities^42^. Similarly, we observed various microtubule-associated abnormalities in *pfdn2*^Δ*10*^ mutant neuroblasts. Different from wild-type neuroblasts that always formed two centrosomes marked by Centrosomin (CNN) during mitosis (Fig. 3D,G; n = 60), in *pfdn2*^Δ*10*^ mutant, 20.5% of neuroblasts contained multiple centrosomes and 6.0% of neuroblasts contained only one centrosome and a monopolar spindle (Fig. 3E,G; n = 83). In addition, 54.2% of *pfdn2*^Δ*10*^ neuroblasts failed to assemble astral microtubules during metaphase (Fig. 3E; n = 83). These microtubule abnormalities in *pfdn2*^Δ*10*^ mutants were exacerbated by the presence of *mgr* mutation. In a *pfdn2*^Δ*10*^ *mgr*^*G5308*^ double mutant, 43.6% of neuroblasts had only one or multiple centrosome during mitosis (Fig. 3F,G; n = 78). In addition, vast majority of neuroblasts lacked obvious astral microtubules in the *pfdn2*^Δ*10*^ *mgr*^*G5308*^ double mutant (Fig. 3F; 89.7%, n = 78). It was shown previously that Mgr depletion led to reduction of both α-tubulin and β-tubulin levels^42^. Similarly, levels of both α-tubulin (Fig. 3H–I; n = 5) and β-tubulin (Fig. 3H,J; n = 5) were dramatically decreased in *pfdn2*^Δ*10*^ larval brains. They were further diminished to 10% and 26% of the wild-type levels in the *pfdn2*^Δ*10*^ *mgr*^*G5308*^ double mutant, respectively (Fig. 3H–J). In contrast, the levels of actin were not dramatically affected in *pfdn2*^Δ*10*^, *mgr*^*-*^ single or *pfdn2*^Δ*10*^ *mgr*^*G5308*^ double mutants (Fig. 3H,K; n = 5). These data suggest that tubulin but not actin level is critically dependent on Prefoldin in the larval brain.

To examine microtubule growth, we depolymerized microtubules with cold treatment in *pfdn2* mutants and monitored microtubule regrowth at 25 °C (Fig. S3E). In wild-type larval brains, we observed that prominent microtubule asters were formed 30 s after the recovery at 25 °C (Fig. S3E; 100%, n = 30) and a normal bipolar spindle was assembled at 120 s in all wild-type neuroblasts (Fig. S3E; 100%, n = 25). In contrast, *pfdn2*^Δ*10*^ mutant neuroblasts displayed much weaker asters after 30 s of recovery (Fig. S3E; 94.1%, n = 34) and only short and disorganized spindles were formed after 120 s of recovery (Fig. S3E; 100%, n = 53). These data suggest that Pfdn2 likely contributes to the microtubule growth through regulating tubulin levels.

### Co-depletion of Pins and Prefoldin leads to massive neuroblast overgrowth

**Figure 4:**
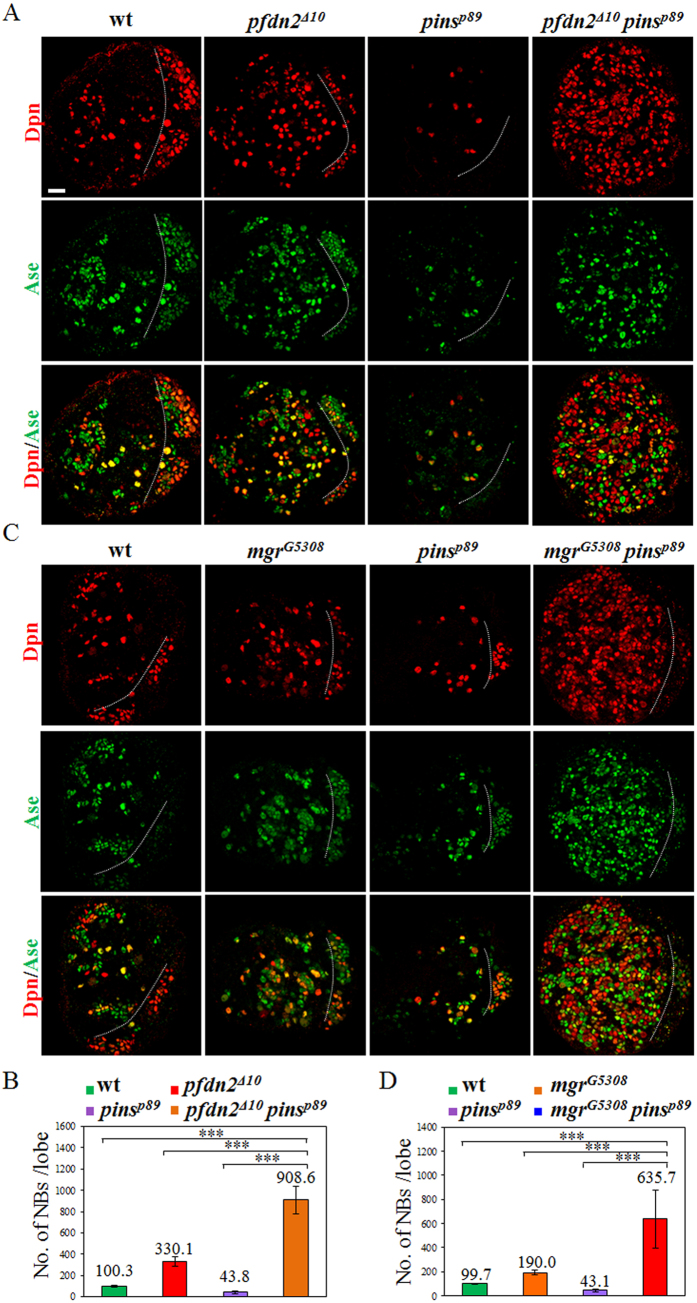
Co-depletion of Prefoldin and Pins results in supernumerary neuroblasts in larval brains. (**A**) Dpn and Ase were labeled in wild-type, *pfdn2*^Δ*10*^, *pins*^*p89*^ and *pfdn2*^Δ*10*^ *pins*^*p89*^ larval brains. (**B**) Quantification of larval brain neuroblasts. (**C**) Dpn and Ase were labeled in wild-type, *mgr*^*G5308*^, *pins*^*p89*^, *mgr*^*G5308*^ *pins*^*p89*^ larval brains. (**D**) Quantification of larval brain neuroblasts. The central brain (CB) is to the left of the white dotted line, which markers the border between the central brain and the optic lobe. ***indicates p < 0.001. Error bars indicate mean standard deviation. Scale bar: 20 μm.

Given that depletion of Pfdn2 or Mgr only resulted in partial loss of Par polarity with intact Pins polarity, we wondered whether Prefoldin functions redundantly with Pins in regulating asymmetric division. Remarkably, simultaneous depletion of Pins and Pfdn2 resulted in severe neuroblast overgrowth in larval brains (Fig. 4A–B). Larval brains that were double mutant for *pfdn2*^Δ*10*^ *pins*^*p89*^ formed 908.6 ± 127.1 neuroblasts per brain hemisphere (Fig. 4A–B; n = 35), while *pfdn2*^Δ*10*^ larval brains had 330.1 ± 43.9 neuroblasts per brain hemisphere (Fig. 4A–B; n = 35). In contrast, *pins*^*p89*^ larval brains developed 43.8 ± 13.4 neuroblasts per brain hemisphere (Fig. 4A–B; n = 25), similar as the previous report^7^,^47^. Most of these ectopic neuroblasts were Dpn^+^ and Ase^−^ (Fig. 4A) or Dpn^+^ PntP1^+^ (Fig. S4A), suggesting that type II neuroblasts are predominant in the population of ectopic neuroblasts. Interestingly, the population of immature INPs which were Dpn^−^ PntP1^+^ appeared to be increased, too (Fig. S4A).

We next examined whether Mgr had a similar synergism with Pins in suppressing neuroblast overgrowth. Indeed, *mgr*^*G5308*^ *pins*^*p89*^ larval brains formed 635.7 ± 238.0 neuroblasts per brain hemisphere (Fig. 4C–D; n = 30), while *mgr*^*G5308*^ homozygous larval brains had 190.0 ± 19.6 neuroblasts per brain lobe (Fig. 4C–D; n = 20). This data strongly suggest the synergism between Pins and Prefoldin in suppressing neuroblast overgrowth.

In contrast, co-depletion of Pfdn2 and aPKC or Par6 suppressed uncontrolled neuroblast proliferation in larval brains. *pfdn2*^Δ*10/01239*^ larval brains formed 283.7 ± 32.9 neuroblasts per brain hemisphere (Fig. S4B–C; n = 16), while knocking down *aPKC* under the *insc-Gal4* formed 89.5 ± 9.0 neuroblasts per brain hemisphere (Fig. S4B–C; n = 20). However, *aPKC* RNAi knockdown partially suppressed neuroblast overgrowth in *pfdn2*^Δ*10/01239*^ larval brains with 174.8 ± 16.9 neuroblasts per brain hemisphere (Fig. S4B–C; n = 22). Likewise, RNAi-mediated knockdown of *par6* in *pfdn2*^Δ*10/01239*^ larval brains resulted in 127.0 ± 22.8 neuroblasts per brain hemisphere (Fig. S4D–E; n = 18), compared to *par6* RNAi knockdown alone (Fig. S4D–E; 83.2 ± 8.2 neuroblasts/lobe, n = 14). These data suggest that the synergism between Prefoldin and Pins in suppressing neuroblast overgrowth is specific.

### Depletion of both Pins and Prefoldin results in symmetric division of neuroblasts

**Figure 5:**
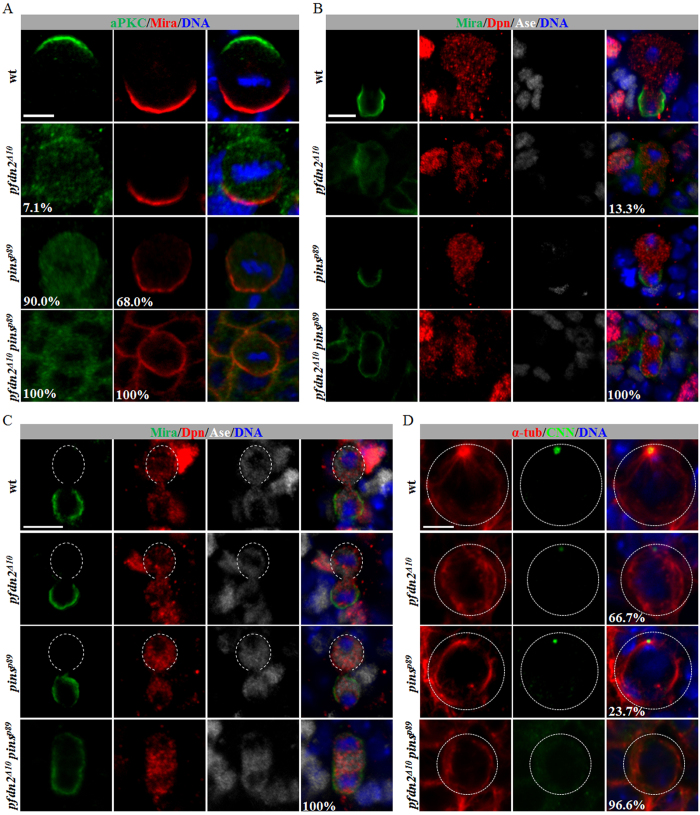
Prefoldin and Pins interact genetically to regulate asymmetric division of neuroblasts and INPs. (**A**) aPKC, Mira and DNA were labeled in wild-type, *pfdn2*^Δ*10*^, *pins*^*p89*^ and *pfdn2*^Δ*10*^ *pins*^*p89*^ larval brains. (**B**–**C**) Dpn, Ase, Mira and DNA were labeled in wild-type, *pfdn2*^Δ*10*^, *pins*^*p89*^ and *pfdn2*^Δ*10*^ *pins*^*p89*^ larval brains. Open white dotted circles indicate INP daughters negative for Mira. (**D**) Wild-type, *pfdn2*^Δ*10*^, *pins*^*p89*^ and *pfdn2*^Δ*10*^ *pins*^*p89*^ larval brains were labeled with CNN, α-tubulin and DNA. Astral microtubules are indicated by white arrows and neuroblasts are outlined by white dotted lines. Scale bar: 5 μm.

Given the synergism observed for Prefoldin and Pins in suppressing neuroblast overgrowth, we examined whether *pfdn2 pins* double mutants had more severe asymmetric division defects than *pfdn2* or *pins* mutant alone. Indeed, asymmetric localization of aPKC and Mira was completely disrupted in *pfdn2*^Δ*10*^ *pins*^*p89*^ neuroblasts at metaphase (Fig. 5A; 100%, n = 50). Moreover, all neuroblasts in *pfdn2*^Δ*10*^ *pins*^*p89*^larval brains underwent equal-size division and segregated Mira into both daughter cells at telophase (Fig. 5B; 100%, n = 30), suggesting that neuroblast symmetric division indeed contributed to the dramatic neuroblast overgrowth. Very interestingly, co-depletion of Pfdn2 and Pins also led to symmetric division of Dpn^+^ Ase^+^ mature INPs. All INPs from wild-type (Fig. 5C; 100%, n = 15), *pins*^*p89*^ (Fig. 5C; 100%, n = 12) or *pfdn2*^Δ*10*^ (Fig. 5C; 100%, n = 20) larval brains divided asymmetrically with Mira segregated into one of the daughter cells. However, all INPs in *pfdn2*^Δ*10*^ *pins*^*p89*^ larval brains divided symmetrically with Mira segregated into both daughter cells (Fig. 5C; 100%, n = 12). These observations suggest that Pfdn2 and Pins function synergistically to regulate asymmetric division of both neuroblasts and INPs.

Interestingly, the α-tubulin levels were further decreased in *pfdn2*^Δ*10*^ *pins*^*p89*^ larval brains, compared to either *pfdn2* or *pins* single mutant (Fig. S5A–B) suggesting that Pins may also have an unexpected function in regulating tubulin levels. To further probe the function of Pins in microtubules, we examined interphase microtubule aster in *pfdn2 pins* double mutant neuroblasts and compared them to *pfdn2* or *pins* single mutant. In 23.7% of the *pins*^*p89*^ neuroblasts, interphase microtubule aster was lost or strongly reduced (Fig. 5D, n = 38) and 66.7% of *pfdn2*^Δ*10*^ interphase neuroblasts failed to form interphase microtubule aster (Fig. 5D, n = 66). Strikingly, 96.6% of *pfdn2*^Δ*10*^ *pins*^*p89*^ neuroblasts failed to form any interphase aster (Fig. 5D; n = 89). Thus, Pfdn2 and Pins function synergistically to regulate microtubule functions, control asymmetric cell division and to suppress the formation of ectopic neuroblasts.

### Depletion of Pfdn2 or Mgr in *pins* mutants induces dedifferentiation of INPs back into neuroblasts

**Figure 6:**
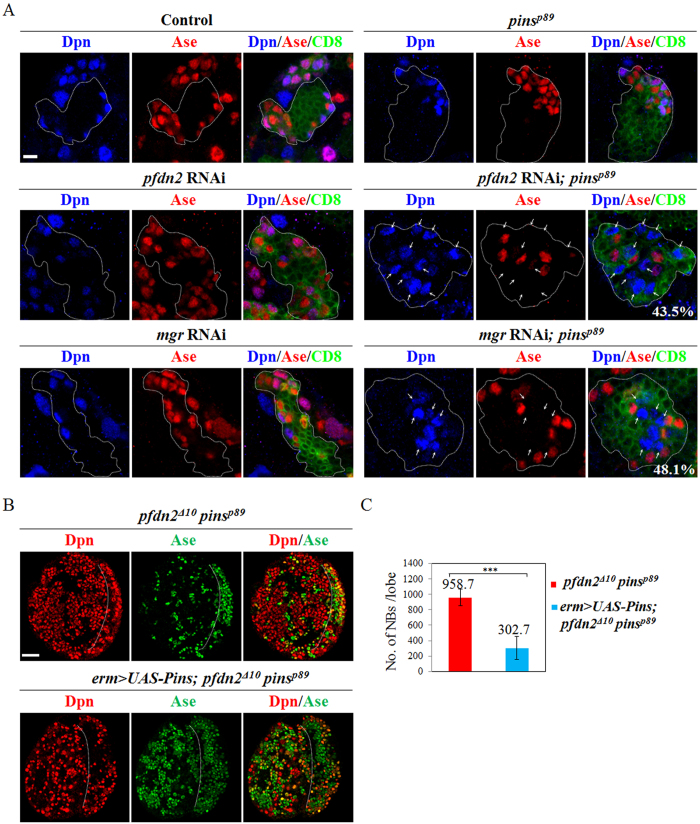
Knockdown of *pfdn2* or *mgr* in *pins* mutant brains results in dedifferentiation of INPs back into neuroblasts. (**A**) Driver control (*erm*-Gal4 UAS-CD8-GFP), *pins*^*p89*^, *pfdn2* RNAi UAS-Dicer2, *pfdn2* RNAi UAS-Dicer2*; pins*^*p89*^, *mgr* RNAi UAS-Dicer2 and *mgr* RNAi UAS-Dicer2*; pins*^*p89*^ larval brains were labeled with Dpn, Ase and CD8. (**B**) *pfdn2*^Δ*10*^ *pins*^*p89*^ and *UAS-Pins pfdn2*^Δ*10*^ *pins*^*p89*^ under *erm*-Gal4 were labeled with Dpn and Ase. The central brain (CB) is to the left of the white dotted line, which markers the border between the CB and the optic lobe. (**C**) Quantification of larval brain neuroblasts. ***indicates p < 0.001. Error bars indicate mean standard deviation. Neuroblasts (NBs, Dpn^+^ Ase^−^ in type II lineages) in the clones are indicated by arrows. Clones are marked by CD8::GFP and outlined by white dotted lines. Scale bars: 5 μm (**A**), 20 μm (**B**).

To explore whether Prefoldin is important for INP function, we knocked down *pfdn2* or *mgr* in *pins* mutant background under an INP-specific driver *erm*-Gal4 (II), which is expressed in Ase^−^ immature INPs. A wild-type INP clone consists of immature INPs (Ase^−^ or Ase^+^), mature INPs (Dpn^+^ Ase^+^), their immediate progeny GMCs (Ase^+^) and neurons (Fig. 6A; 100%, n = 50). RNAi-mediated knockdown of *pfdn2* (Fig. 6A; 100%, n = 50) under *erm*-Gal4 resembled wild-type INP clones. Surprisingly, *pfdn2* RNAi knockdown in *pins*^*p89*^ mutants resulted in numerous aberrant Dpn^+^ Ase^-^ type II neuroblasts (Fig. 6A; 43.5%, n = 46), while INP clones in *pins*^*p89*^ mutants alone did not contain any type II neuroblasts (Fig. 6A; 100%, n = 30). This observation suggests that upon depletion both Pfdn2 and Pins, INPs undergo dedifferentiation back into neuroblasts. Consistently, *pins*^*p89*^ mutant with *mgr* RNAi knockdown resulted in similar dedifferentiation phenotype (Fig. 6A; 48.1%, n = 52; S6; 41.7%, n = 24), while *mgr* knockdown alone in INPs did not cause any phenotype (Fig. 6A; n = 42; S6; n = 25). These data suggest that co-depletion of Prefoldin and Pins induces dedifferentiation of INPs back into neuroblasts. The severe brain overgrowth observed in *pfdn2*^Δ*10*^ *pins*^*p89*^ larval brains (Fig. 6B–C; 958.7 ± 107.0 neuroblasts/lobe, n = 15) can be partially reversed by overexpressing a wild-type *pins* under the INP specific driver *erm*-Gal4 (Fig. 6B–C; 302.7 ± 148.9 neuroblasts/lobe, n = 16), suggesting that Pins indeed functions to suppress INP dedifferentiation.

### Knockdown of either *α-tubulin* or *β-tubulin* in *pins* mutants leads to massive neuroblast overgrowth

**Figure 7:**
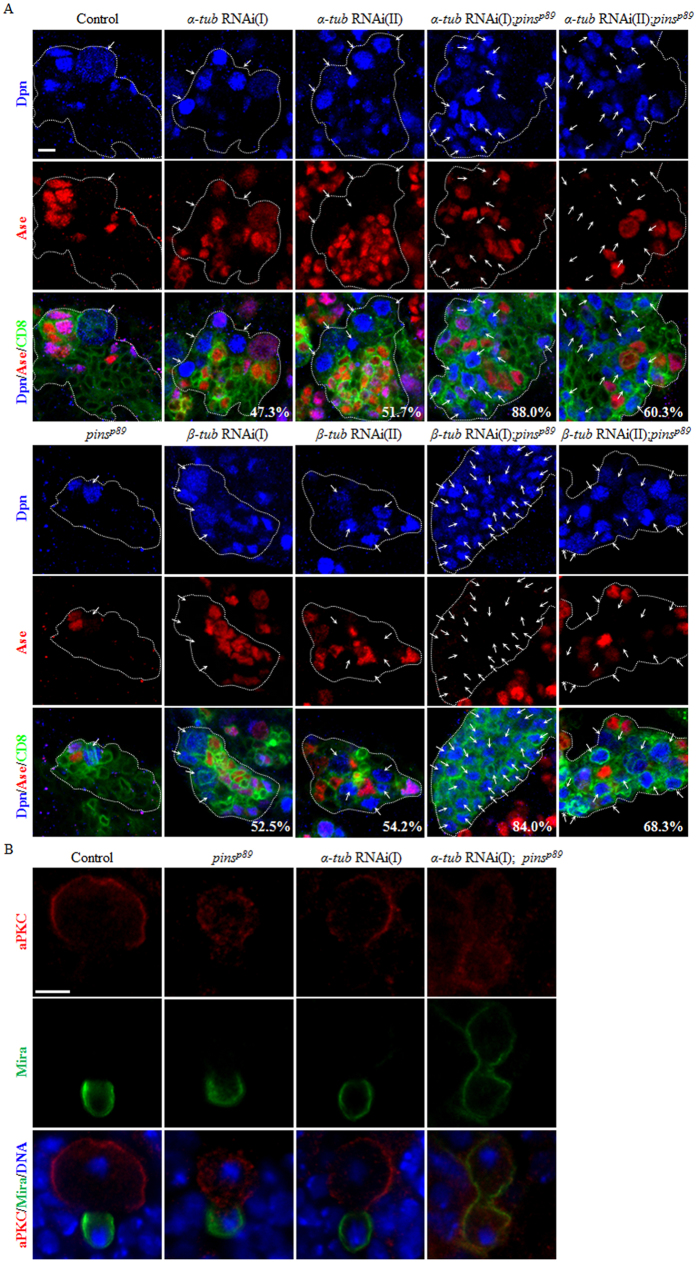
Reduction of tubulin levels in *pins* mutant brains results in neuroblast overgrowth and asymmetric division defects. (**A**) Driver control (*insc*-Gal4 UAS-CD8-GFP), *pins*^*p89*^, *α-tub* RNAi(I), *α-tub* RNAi(II), *α-tub* RNAi(I); *pins*^*p89*^, *α-tub* RNAi(II); *pins*^*p89*^, *β-tub* RNAi(I), *β-tub* RNAi(II), *β-tub* RNAi(I)*; pins*^*p89*^ and *β-tub* RNAi(II)*; pins*^*p89*^ were labeled with Dpn, Ase and CD8. (**B**) Driver control (*insc*-Gal4), *pins*^*p89*^, *α-tub* RNAi(I) and *α-tub* RNAi(I)*; pins*^*p89*^ larval brains were labeled with aPKC, Mira and DNA. Type II neuroblasts (Dpn^+^ Ase^-^) in the clones are indicated by arrows. Clones are marked by CD8::GFP and outlined by white dotted lines. Scale bar: 5 μm.

Given that Prefoldin and Pins synergistically regulate tubulin level in larval brains, we determined whether a decrease of microtubule function in *pins* mutant background could mimic the *pins pfdn2* double mutants. First, we knocked down various *α*-*tubulin* and *β-tubulin* in neuroblasts using *insc*-Gal4. *α-tubulin* RNAi knockdown resulted in ectopic neuroblasts in both type I lineages (Fig. S7A; *α-tub* RNAi(I)/*α-tub67C/CG8308,* 45.5%, n = 66 and *α-tub* RNAi(II)/*α-tub84B/CG1913*, 53.3%, n = 60) and type II lineages (Fig. 7A; α-tu*b* RNAi(I), 47.3%, n = 55 and *α-tub* RNAi(II), 51.7%, n = 60). Likewise, *β-tubulin* RNAi knockdown led to generation of ectopic neuroblasts in both type I neuroblast lineages (Fig. S7A; *β-tub* RNAi(I)/*β-tub60D*/*CG3401*, 47.5%, n = 40 and *β-tub* RNAi(II)/*β-tub56D*/*CG9277*, 52.1%, n = 48) and type II neuroblast lineages (Fig. 7A; *β-tub* RNAi(I), 52.5%, n = 40 and *β-tub* RNAi(II), 54.2%, n = 48). Next, we knocked down *α-tubulin* in *pins*^*p89*^ mutant background. Surprisingly, this led to enormous overgrowth in neuroblast lineages (Fig. 7A; *α-tub* RNAi(I) *pins*^*p89*^, 88.0%, n = 50; *α-tub* RNAi(II) *pins*^*p89*^, 60.3%, n = 58), mimicking the phenotypes in *pins pfdn2* double mutant. Consistently, *β-tubulin* knockdown in *pins*^*p89*^ mutants also caused dramatic neuroblast overgrowth (Fig. 7A; *β-tub* RNAi(I) *pins*^*p89*^, 84.0%, n = 50; *β-tub* RNAi(II) *pins*^*p89*^, 68.3%, n = 41). Both *α-tubulin and β-tubulin* knockdown in these experiments were evident, as tubulin levels were dramatically reduced in these clones (Fig. S7B–E). Next, we examined whether this massive neuroblast overproliferation are attributed by asymmetric division defects. In wild-type prometa/metaphase neuroblasts, aPKC formed an apical crescent, while Mira accumulated at the basal cortex (Fig. S7F; 100%, n = 50). This asymmetric localization was also observed in most of the neuroblasts with *α-tubulin* knockdown alone (Fig. S7F; 98.4%, n = 63). However, *pins*^*p89*^ mutant with *α-tubulin* RNAi further exacerbated delocalization of both aPKC (Fig. S7F; 96.7%, n = 30) and Mira (Fig. S7F; 86.7%, n = 30) compare to *pins*^*p89*^ mutants alone (Fig. S7F; aPKC, 92.0%, n = 25; Mira, 65.2%, n = 23). In wild-type neuroblasts, apical aPKC and basal Mira were segregated exclusively into one of the daughter cells at telophase (Fig. 7B; 100%, n = 25). Asymmetric segregation of aPKC and Mira was also observed in *pins*^*p89*^ mutant (Fig. 7B; 100%, n = 15) and *α-tubulin* RNAi knockdown neuroblasts (Fig. 7B; 97.7%, n = 44), presumably through “telophase rescue”. However, mis-segregation of both aPKC and Mira at telophase occurred in most of *pins*^*p89*^ mutant neuroblasts with *α-tubulin* RNAi knockdown (Fig. 7B; 73.1%, n = 26). These observations suggest that co-depletion of tubulin and Pins results in severe defects of asymmetric division at both metaphase and telophase, which contributes to the severe overgrowth phenotype.

### Knockdown of either *α-tubulin* or *β-tubulin* in *pins* mutants leads to dedifferentiation of INPs back into neuroblasts

**Figure 8:**
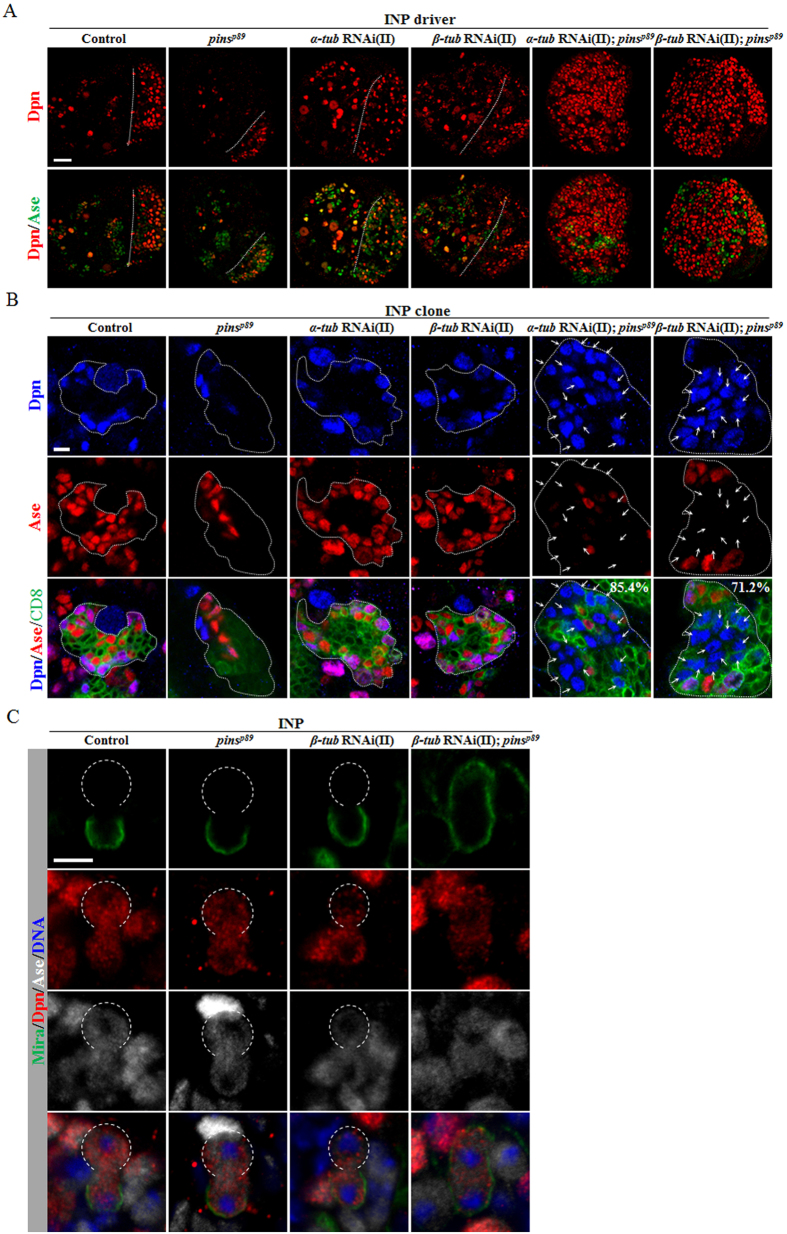
Reduction of tubulin levels in *pins* mutant brains results in dedifferentiation of INPs back into neuroblasts. (**A**) Dpn and Ase were labeled in driver control (*erm*-Gal4 UAS-Dicer2), *pins*^*p89*^*, α-tub* RNAi(II)*, β-tub* RNAi(II), *α-tub* RNAi(II); *pins*^*p89*^, and *β-tub* RNAi(II)*; pins*^*p89*^ larval brains. The central brain (CB) is to the left of the white dotted line, which markers the border between the CB and the optic lobe. (**B**) Driver control (*erm*-Gal4 UAS-CD8-GFP), *pins*^*p89*^*, α-tub* RNAi(II), *β-tub* RNAi(II), *α-tub* RNAi(II); *pins*^*p89*^, and *β-tub* RNAi(II)*; pins*^*p89*^ larval brains were labeled with Dpn, Ase and CD8. Type II neuroblasts (Dpn^+^ Ase^-^) in the clones are indicated by arrows. Clones are marked by CD8::GFP and outlined by white dotted lines. (**C**) Driver control (*erm*-Gal4 UAS-Dicer2), *pins*^*p89*^, *β-tub* RNAi(II) and *β-tub* RNAi(II)*; pins*^*p89*^ larval brains were labeled with Dpn, Ase, Mira and DNA. Open white dotted circles indicate INP daughters negative for Mira. Scale bars: 20 μm (**A**), 5 μm (**B**,**C**).

Next, we wondered whether tubulins also play a role in INPs to prevent dedifferentiation. Toward this end, we used *erm*-Gal4 to knock down *α-* or *β-tubulin* in INPs of *pins* mutants. Control brains formed 100.2 ± 8.2 neuroblasts per brain hemisphere (Fig. 8A, S8A; n = 15), and knockdown of *α-tubulin* under the *erm*-Gal4 gave rise to 110.3 ± 9.8 neuroblasts per brain hemisphere (Fig. 8A, S8A; n = 20), which was similar to that of the control brains. Remarkably, knockdown of *α-tubulin* under the control of *erm*-Gal4 in *pins*^*p89*^ mutants caused severe neuroblast overproliferation (Fig. 8A, S8A; 438.7 ± 320.1 neuroblasts/lobe, n = 21), in contrast to *pins*^*p89*^ larval brains that formed fewer neuroblasts (Fig. 8A, S8A; 45.5 ± 13.2 neuroblasts/lobe, n = 20). These data suggest that co-depletion of α-tubulin and Pins specifically in INPs results in ectopic neuroblasts due to INP dedifferentiation. Likewise, we observed the similar massive neuroblast overgrowth phenotype in *pins*^*p89*^ larval brains with *β-tubulin* knockdown (Fig. 8A, S8A; 520.3 ± 405.2 neuroblasts/lobe, n = 18), while *β-tubulin* knockdown under *erm*-Gal4 alone did not result in obvious neuroblast overgrowth (Fig. 8A, S8A; 115.5 ± 10.6 neuroblasts/lobe, n = 25). Next, we examined single INP clones in these mutants. None of the INP clones in control (Fig. 8B; 100%, n = 30), *pins*^*p89*^(Fig. 8B; 100%, n = 25), *α-tubulin* RNAi knockdown (Fig. 8B; 100%, n = 35), or *β-tubulin* RNAi knockdown (Fig. 8B; 100%, n = 35) larval brains contained any Dpn^+^ Ase^-^ type II neuroblast. However, *pins*^*p89*^ larval brains with *α-tubulin* knockdown produced ectopic type II neuroblasts in 85.4% of the INP clones (Fig. 8B; n = 41). Similarly, *β-tubulin* knockdown in *pins*^*p89*^mutants displayed excess neuroblasts in 71.2% of the INP clones (Fig. 8B; n = 59). These data suggest that tubulins and Pins function synergistically in suppressing dedifferentiation of INPs back into neural stem cells.

### Knockdown of *tubulin* in *pins* mutant results in symmetric division of INPs

To assess whether *tubulin* knockdown in *pins* mutant affects asymmetric division of INPs, we examined the localization of Mira, which is asymmetrically localized in wild-type INPs at prometa/metaphase (Fig. S8B; 100%, n = 30). *β-tubulin* knockdown alone under *erm*-Gal4 did not influence the asymmetric localization of Mira (Fig. S8B; 100%, n = 26). In *pins*^*p89*^ mutants, Mira was delocalized in 87.1% of the INPs (Fig. S8B; n = 31), and this phenotype was further enhanced with *β-tubulin* knockdown in INPs (Fig. S8B; 93.8%, n = 32). Next, we examined whether co-depletion of tubulin and Pins caused asymmetric division defects of INPs at telophase. Similar to wild-type INPs (Fig. 8C; 100%, n = 20), all INPs from *pins*^*p89*^ (Fig. 8C; 100%, n = 10) and INP-specific knockdown of *β-tubulin* (Fig. 8C; 100%, n = 15) brains divided asymmetrically with Mira exclusively segregated to one of the daughter cells. However, 90.0% of the INPs in *pins*^*p89*^ larval brains with *β-tubulin* knockdown in INPs divided symmetrically and mis-segregated Mira into both daughter cells (Fig. 8C; n = 20). These observations suggest that tubulins and Pins function synergistically to regulate asymmetric division of INPs.

### Overexpression of α-tubulin suppresses various defects associated with *prefoldin pins* double mutants

Given that defects observed in *pfdn2 pins* double mutants can be phenocopied by co-depleting tubulin and Pins, we investigated whether restoring tubulin levels in *pfdn2 pins* double mutants is sufficient to prevent neuroblast overgrowth. Overexpression of GFP-α-tubulin under the ubiquitin promoter did not lead to any obvious changes to neuroblast number (Fig. S9A–C). In contrast to 866.1 ± 138.6 neuroblasts per brain hemisphere in *pfdn2*^Δ*10*^ *pins*^*p89*^ mutant brains (Fig. S9A–B; n = 20), there were only 123.2 ± 49.4 neuroblasts per brain hemisphere in *pfdn2*^Δ*10*^ *pins*^*p89*^double mutant brains expressing GFP-α-tubulin (Fig. S9A–B; n = 23). This observation suggests that the overexpression of GFP-α-tubulin dramatically suppresses the unrestrained neuroblast proliferation in *pfdn2*^Δ*10*^ *pins*^*p89*^ larval brains. We ascertained whether this suppression was partially contributed by the restoration of asymmetric division. Indeed, the polarity of Baz (Fig. S9D; 33.3%, n = 21), aPKC (Fig. S9D; 18.8%, n = 32), Numb (Fig. S9D; 37.5%, n = 40) and Mira (Fig. S9D; 43.8%, n = 32) in prometa/metaphase neuroblasts were partially restored. Moreover, asymmetric segregation of aPKC and Mira was rescued in 66.7% of the neuroblasts at telophase with GFP-α-tubulin expression (Fig. S9E; n = 24), in contrast to a complete disruption in *pfdn2*^Δ*10*^ *pins*^*p89*^ mutant brains (Fig. S9E; 100%, n = 20). These data suggest that Pfdn2 and Pins suppress neuroblast overgrowth through regulating tubulins levels.

## Discussion

Here we have identified an unexpected synergism between Prefoldin and Pins in suppressing neuroblasts overgrowth (Fig. S9F). We show that various subunits of Prefoldin complex are implicated in asymmetric division of neuroblasts, especially during asymmetric protein segregation at telophase. It is known that depletion of Pins results in the formation of smaller larval brains, despite partial loss of neuroblasts polarity^7^,^47^. Interestingly, co-depletion of Pfdn2 and Pins results in severe neuroblasts overgrowth, while Pfdn2 depletion alone only causes mild brain overgrowth. This phenotype is contributed by a combination of loss of neuroblast polarity, defects of asymmetric division of INPs, as well as INP dedifferentiation. Knocking down *tubulins* in *pins* mutant background mimics the co-depletion of Prefoldin and Pins, suggesting that tubulin stability appears to be critical for the suppression of neuroblast overgrowth in the absence of Pins function. Our data also suggest that Prefoldin function and tubulin stability in INPs are important to suppress their dedifferentiation back into neuroblasts.

How microtubules induce cortical polarity is poorly understood in *Drosophila* neuroblasts. Previously, one report showed that kinesin Khc-73, which localized at the plus end of astral microtubules, and Discs large (Dlg) induced cortical polarization of Pins/Gαi in neuroblasts^48^. However, microtubules are considered not essential for neuroblast polarity^49^,^50^. Here, we show that *Drosophila* Prefoldin regulates asymmetric division of both neuroblasts and INPs through tubulins, suggesting an important role of microtubules in neuroblast polarity. The essential role of microtubules directly regulating cell polarity is found in various systems. During *C. elegans* meiosis, a microtubule-organizing center is necessary and sufficient for the establishment of the anterior-posterior polarity^51^. In the fission yeast *Schizosaccharomyces pombe*, interphase microtubules directly regulate cell polarity through proteins such as tea1p^52^. In mammalian airway cilia, microtubules are required for asymmetric localization of planer cell polarity proteins^53^.

We show that the role of *Drosophila* Prefoldin complex in regulating asymmetric division is very likely dependent on microtubules. This is consistent with the known essential role of Prefoldin for maintaining tubulin levels in various organisms such as yeast, *C. elegans*, plants and mammals^37^,^40^,^54^,^55^. In yeast, Gim (Prefoldin) null mutants become super-sensitive to the microtubule-depolymerizing drug benomyl as a result of a reduced level of α-tubulin^37^. In the absence of Prefoldin, the function of the chaperone pathway is damaged and unable to fold sufficient amount of tubulins for normal yeast growth^36^. In *C. elegans*, reducing Prefoldin function causes defects in cell division presumably due to the reduction of tubulin levels and microtubule growth rate^40^. Genetic analysis of mammalian Prefoldin also suggests that cytoskeletal proteins like actin and tubulin make up the major substrate of Prefoldin in mammals^41^. These studies in different organisms together suggest that Prefoldin complex plays a conserved central role in tubulin folding.

“Telophase rescue”, a term refers to the phenomenon that protein mis-localization at metaphase is completely restored at telophase, is observed in many mutants that affect neuroblast asymmetric division^45^. However, both apical and basal proteins are still mis-segregated in *pfdn2* and *mgr* mutants, suggesting that “telophase rescue” is defective in these mutants. Telophase rescue is regulated by TNF receptor-associated factor (DTRAF1), which binds to Baz and acts downstream of Egr/TNF^56^. Telophase rescue also depends on Worniu/Escargot/Snail family proteins and a microtubule-dependent Khc-73/Dlg pathway^48^,^57^. Pins did not form a protein complex with Mgr, α-tubulin or β-tubulin in co-immunoprecipitation assays (Fig. S10). Given that Dlg is a Pins-interacting protein^58^, Prefoldin appears to function in a different pathway with Dlg or Khc-73 during asymmetric division.

Recently, *merry-go-round* (*mgr*), encoding Prefoldin 3 (Pfdn3)/VBP1/Gim2 subunit, was reported to regulate spindle assembly^42^. Loss of *mgr* led to formation of monopolar mitotic spindles and loss of centrosomes because of improper folding and destabilization of tubulins^42^. Our analysis on Pfdn2 indicates that *pfdn2* mutants displayed similar spindle and centrosome abnormalities. In addition, the incorrectly folded tubulin due to loss of *mgr* may be eliminated by *Drosophila* von Hippel Lindau protein (Vhl), an E3 ubiquitin-protein ligase^42^. Interestingly, our data suggest that Prefoldin has a tumor-suppressor like function in preventing neuroblast overgrowth. However, *Drosophila* Vhl is not important for brain tumor suppression, as its loss-of-function neither affects number of neuroblasts nor suppresses overgrowth observed in *pfdn2* RNAi or *mgr* RNAi (data not shown).

We show a novel synergism between Prefoldin and Pins in suppressing dedifferentiation of INPs back into neuroblasts. Prefoldin and Pins apparently suppress dedifferentiation through regulating tubulin levels. It is likely that appropriate tubulin levels in INPs are important for their differentiation, while reducing tubulin levels can increase the risk of INP dedifferentiation. Currently, several cell fate determinants such as Brat, Numb and the SWI/SNF chromatin remodeling complex with its cofactors Erm and Hdac3 are critical to suppress INP dedifferentiation back into neuroblast^29^,^32^,^33^,^34^,^35^. It is currently unknown whether or how Prefoldin/Pins are linked to these known suppressors of dedifferentiation. It is possible that symmetric division of INPs causes reduced levels of Brat and Numb in these abnormal INP daughters, leading to their dedifferentiation. Alternatively, Prefoldin might regulate transcription of genes within INPs to suppress dedifferentiation. It was reported that the human homolog of Pfdn5, MM-1, has a role in transcriptional regulation by binding to the E-box domain of c-Myc and represses E-box-dependent transcriptional activity^59^. Interestingly, Prefoldin Subunit 5 gene is deleted in Canine mammary tumors, suggesting that it may be a tumor suppressor gene^60^. Our study has revealed a novel mechanism by which Prefoldin and Pins function through tubulin stability to suppress stem cell overgrowth. It is expected to contribute to the understanding of mammalian/human Prefoldin function in tumorigenesis.

## Materials and Methods

### Fly stocks and genetics

The fly strains used in this paper were: *pfdn2*^Δ*10*^, *pfdn2*^Δ*17*^, UAS-Pfdn2, UAS-Pfdn2-Venus, *pins*^*p89*^(F. Yu), type II neuroblast driver (w; UAS-Dicer2, wor-Gal4, ase-Gal80/CyO; UAS-mCD8-GFP/TM3, Ser; J. Knoblich), neuroblast driver (*insc*-Gal4; J. Knoblich), INP driver (*erm*-Gal4/CyO; GM Rubin), pUbiquitin-α-tub-GFP (Gonzalez, C.). The following stocks were obtained from Bloomington *Drosophila* Stock Center (BDSC): *pfdn2*^*01239*^(BDSC#11526), *mgr*^*G5308*^ (BDSC#30151), *Df(3L)BSC457* (BDSC#24961), *Df(3R)Exel6160* (BDSC#7639). The following RNAi stocks were obtained from Vienna Drosophila Resource Center (VDRC): *pfdn2* RNAi (v28794/*CG6302*), *pfdn1* RNAi (v18210/*CG13993*), *mgr* RNAi (v27727/*CG6719*), *pfdn4* RNAi (v46220/*CG10635*), *pfdn5* RNAi (v29812/*CG7048*) *pfdn6* RNAi (v34204/*CG7770*), *α-tubulin* RNAi(I) (v24783/*α-tub67C/CG8308), α-tubulin* RNAi(II) (v52345/*α-tub84B/CG1913)*, *β-tubulin* RNAi(I) (v104937/*β-tub60D*/*CG3401)*, *β-tubulin* RNAi(II) (v24138/*β-tub56D*/*CG9277)*, *par6* RNAi (v19732/*CG5884*), and *aPKC* RNAi (v2907/*CG42783*).

### Generation of *pfdn2* ^Δ*10*^ and *pfdn2* ^Δ*17*^

*pfdn2*^Δ*10*^ and *pfdn2*^Δ*17*^were generated by imprecise excision of *P{EPgy2}l(3)01239*^*EY06124*^ (BDSC#19918), a P element inserted at the 11^th^ base pair from the *pfdn2* transcription start site. Deletions were verified by PCR amplification and sequencing. Oligos used were: 5′-TGTGCAAGGCTGTTTCTCAC-3′ (forward) and 5′-TTATGTTTAAGTAACTGAAGTTGTGCT-3′ (reverse).

### Molecular cloning

The full-length cDNAs of *pfdn2*, *mgr*, and *pfdn5* were amplified from cDNA clones obtained from *Drosophila* Genomics Resources Center (DGRC) and sub-cloned into Gateway^®^ pENTR^TM^ vector (pENTR™⁄D-TOPO^®^ Cloning Kit, Invitrogen). Myc or Flag tags were added into the N-terminus of the gene sequences by LR recombination reactions (Gateway^®^ LR Clonase^®^ II Enzyme mix, Invitrogen) using pAMW or pAFW destination vectors respectively. The oligos that were used to amplify various DNA fragments were listed in Table 1.

### Transgenic flies

To generate pUAS-Pfdn2-Venus or pUAS-Pfdn2 construct, the full-length coding region of Pfdn2 was tagged with (or without) Venus at the C-terminus by the LR recombination between pENTRY *pfdn2* and pTWV (or pTW) vector. Briefly, using Gateway LR Clonase II enzyme mix, the full-length coding gene of Pfdn2 from the entry construct was transferred into the Gateway destination vector pTWV (or pTW). UAS-Pfdn2-Venus or UAS-Pfdn2 transgenic flies were generated by standard P element-mediated transformation of pUAS-Pfdn2-Venus or pUAS-Pfdn2 by BestGenes, Inc. Oligos used for sequencing were: 5′-TATAAATAGAGGCGCTTCGT-3′ (forward) and 5′-CTTCGGGCATGGCGGACTTG-3′ (reverse).

### Clonal analysis

FRT2A *pfdn2*^Δ*10*^*/TM6B* and *elav-Gal4 hsFlpase; UAS-nLacZ UAS-CD8:: GFP/CyO;FRT2A tubP-Gal80* (MARCM driver) flies were used to generate *pfdn2* mutant clones. Larvae were heat-shocked twice at 37 °C for 2 hrs, at 24 h ALH and 40 h ALH respectively, and were allowed to develop for another 3 days at 25 °C. RNAi knockdown was carried out in 29 °C for 4 days after egg-laying.

### Immunohistochemisry

Larval brain dissection and immunostaining were carried out essentially as described previously^25^. Briefly, larval brains were dissected and fixed in 3.7% formaldehyde in PBS with 0.3% Triton X-100. Fixed brains were washed for three times (10 min each) before one hour-blocking with 3% BSA. Larval brains were then incubated with primary antibodies over night at 4 °C. Secondary antibodies were then added into the samples followed by three times washing with PBT. After incubated with ToPro-3 to stain DNA for 20 min, larval brains were mounted in Vectorshield (Vector Laboratory). Images were obtained using Zeiss LSM 710 confocal microscope and processed with Adobe Photoshop CS5.

### Western blotting

Third-instar larval brains were dissected in PBS and homogenized in RIPA buffer (50 mM Tris HCl pH 7.5, 150 mM NaCl, 1 mM EDTA, 1% Triton X-100, 0.5% sodium deoxycholate, 0.1% SDS). Western blotting was carried out according to standard procedures.

### Antibodies

The following primary antibodies were used: guinea pig anti-Dpn [J. Skeath, immunofluorescence (IF) 1:1000], rabbit anti-Ase (YN Jan, IF 1:500), mouse anti-Mira (F. Matsuzaki, IF 1:80), rat anti-CD8 (Life technologies, IF 1:200), rabbit anti-aPKCζ C20 (Santa Cruz Biotechnologies, IF 1:200, western blot (WB) 1:3000), guinea pig anti-Bazooka (F. Yu, IF 1:500, WB 1:1000), guinea pig anti-Gαi (F. Yu, IF 1:200), rabbit anti-Par6 (J. Knoblich, IF 1:200, WB 1:1000), rabbit anti-Pon (Y.N. Jan, IF 1:200), rabbit anti-Pins (F. Yu, IF 1:200, WB 1:3000), mouse anti-α-tubulin (Sigma, IF 1:100, WB 1:5000), mouse anti-β-tubulin (DSHB, IF 1:50, WB 1:25), rabbit anti-CNN (E. Schejter, IF 1:1000), rabbit anti-PntP1 (J. Skeath, IF 1:500), mouse anti-Actin (MP Biomedicals, WB 1:5000), rabbit anti-Pfdn2 (this study, IF 1:500, WB 1:1000), mouse anti-Myc (Abcam, WB 1:3000), mouse anti-Flag (Sigma, WB 1:3000).

### Generation of anti-Pfdn2 antibody

The cDNA region encoding the full length of Pfdn2 was amplified by PCR and subsequently cloned into *EcoRI* and *SalI* sites of PAML-C2-X vector, using the In-Fusion HD cloning Kit (Clontech). The primers used were: 5′-AGGATTTCAGAATTCATGAGCACCGAATCGGCGAAG-3′ (forward), 5′-TTGCCTGCAGGTCGATCAGTTGAACACCAGGACATT-3′ (reverse). The expression of MBP-Pfdn2 was induced by isopropyl β-D-1-thiogalactopyranoside (IPTG) and purified using glutathione-Sepharose (GE Healthcare) and eluted with glutathione. MBP-Pfdn2 was injected into one rabbit and purified by GenScript (Hong Kong).

### S2 cell culture, transient transfection and co-immunoprecipitation

*Drosophila* S2 cells were cultured in Shields and Sang m3 insect medium (Sigma-Aldich) with 10% fetal bovine serum (Hyclone) at 25 °C. Plasmids were transfected into S2 cells using Effectene Transfectin Reagent (QIAGEN). S2 cells were harvested 48 h after transfection and homogenized with lysis buffer (25 mM Tris pH8, 27.5 mM NaCl, 20 mM KCl, 25 mM sucrose, 10 mM EDTA, 10 Mm EGTA, 1 mM DTT, 10% (v/v) glycerol, 0.5% Nonidet P40) supplemented with Proteases inhibitors (Boeringher). Supernatants were collected and imunoprecipitated with mouse anti-Flag or anti-Myc for overnight at 4 °C, followed by incubation with Protein A/G beads (Pierces) for two hours at 4 °C. Bound proteins were analyzed by western blotting after washing for three times with cold PBS.

### Quantification of spindle orientation

The apico-basal polarity of metaphase neuroblasts was indicated by a line perpendicular to the apical protein crescent, while the spindle axis was labeled by a second line parallel with the α-tubulin labeled mitotic spindle. The angles between these two lines were measured in metaphase neuroblasts.

### EdU labeling

EdU labeling was processed with the Click-iT EdU Imaging Kits (Invetrogen). Briefly, third instar larval brains were dissected in PBS and incubated with 10 uM EdU working solution (Invetrogen) for 45 minutes. The brains were dissected and fixed in 3.7% formaldehyde with PBS for 15 mins at room temperature and permeabilized with 0.3% TritonX-100 in PBS. Incorporated EdU was detected after incubation with the Click-iT reaction cocktail (1 × Click-iT reaction buffer, 4% CuSO_4_, 0.24% Alexa Fluor azide, 10% Reaction buffer additive).

### Microtubule regrowth assay

Larval brains were dissected in Shields and Sang m3 insect medium (Sigma-Aldich) with 10% fetal bovine serum (Hyclone) and incubated on ice for 30 min to depolymerize microtubules (MTs) completely. After that, brain samples were incubated in a water bath at 25 °C at various time points (0 s, 30 s, and 120 s) to induce MTs regrowth. Samples were fixed in testis buffer (TB: 183 mM KCl, 47 mM NaCl, 10 mM Tris, and 1 mM EDTA, pH6.8) supplemented with 37% formaldehyde.

## Additional Information

**How to cite this article**: Zhang, Y. *et al.* Prefoldin and Pins synergistically regulate asymmetric division and suppress dedifferentiation. *Sci. Rep.* **6**, 23735; doi: 10.1038/srep23735 (2016).

## Supplementary Material

Supplementary Information

## Figures and Tables

**Table 1:** List of oligos used to amplify the cDNA of Prefoldin subunits.

Gene name	Oligo name	Oligo sequence (5′–3′)
*pfdn2/CG6302*	*CG6302* F	CACCATGAGCACCGAATCGGCGAAGCCGGCA
	*CG6302* R	GTTGAACACCAGGACATTACGGTTCTCCGC
*mgr/CG6719*	*CG6719* F	CACCATGACAGGAATAATGGACTCGGTG
	*CG6719* R	CACCATGCCCAAAATGGACAACGAG
*pfdn5/CG7048*	*CG7048* F	CACC ATGGCTGCCACCCCAA
	*CG7048* R	CTAGGAGCTCTGGGTAACC
