# Supplementary material for: Prefoldin and Pins synergistically regulate asymmetric division and suppress dedifferentiation
Source: Sci Rep. 2016 Mar 30;6:23735. doi: 10.1038/srep23735 (PMC4812327; doi:10.1038/srep23735)
Supplement: Supplementary Information [file srep23735-s1.pdf]

Prefoldin and Pins synergistically regulate asymmetric division and suppress dedifferentiation

Yingjie Zhang[1,2], Madhulika Rai[3], Cheng Wang[1], Cayetano Gonzalez[3,4] and Hongyan Wang[1,2,5]*

Supplementary Figure 1

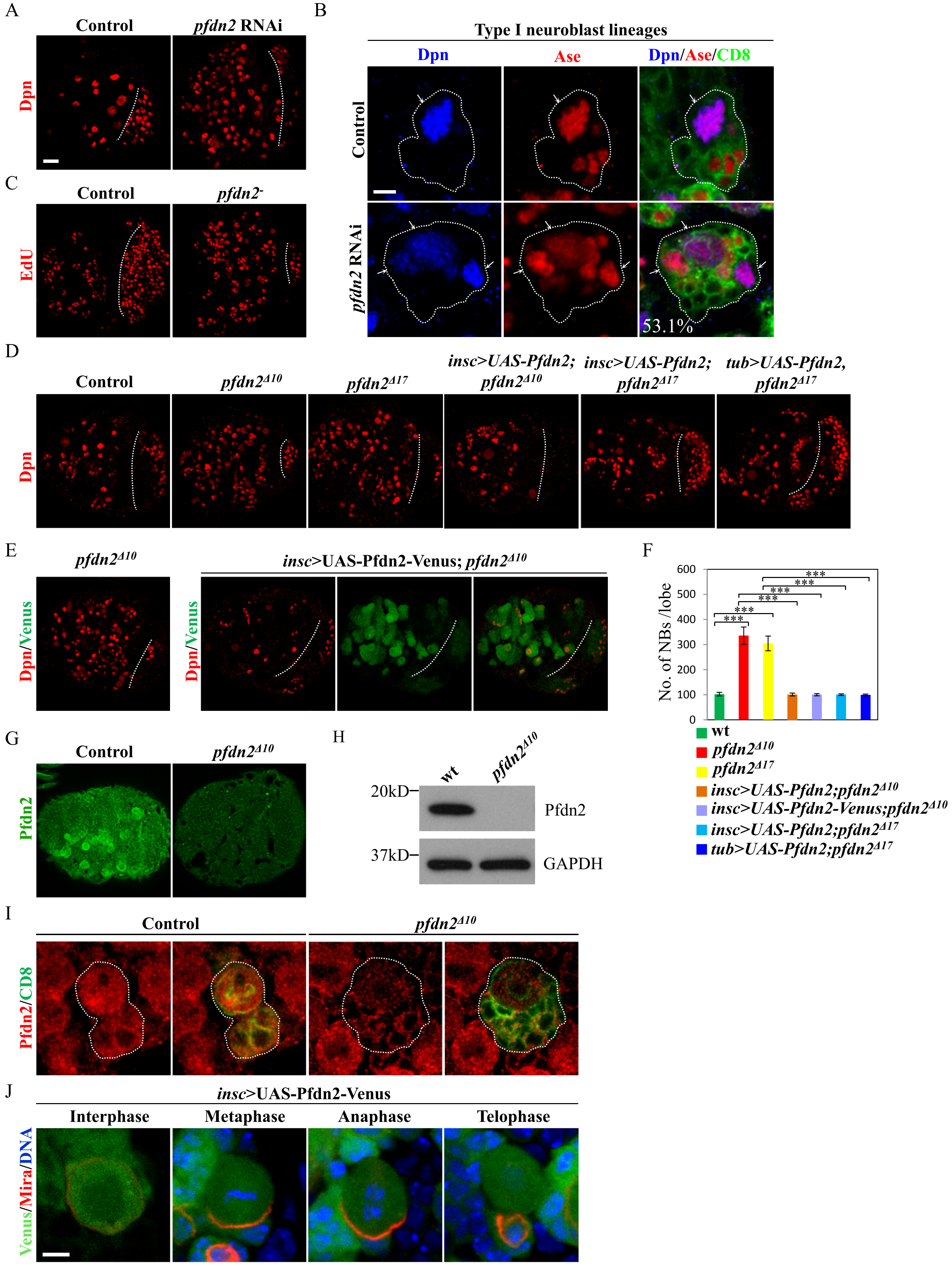

Supplementary Figure 1. Pfdn2 suppresses neuroblast overproliferation in larval brains. (A) Driver control (*insc*-Gal4 UAS-Dicer2) and *pfdn2* RNAi were labeled with Dpn. (B) Driver control (*insc*-Gal4 UAS-CD8-GFP UAS-Dicer2) and *pfdn2* RNAi were labeled with Dpn, Ase and CD8. Neuroblasts (Dpn$^+$ Ase$^+$ in type I lineages) in the clones are indicated by arrows. Clones are marked by CD8::GFP and outlined by white dotted lines. (C) Wild-type and *pfdn2*$^-$ [*pfdn2*^{01239}*/Df(3L)BSC457*] larval brains were labeled with EdU (5-ethynyl-2′-deoxyuridine). (D) Larval brains in wild-type, *pfdn2*$^{\Delta 10}$, *pfdn2*$^{\Delta 17}$, *insc*-Gal4>UAS-Pfdn2 *pfdn2*$^{\Delta 10}$, *insc*-Gal4>UAS-Pfdn2 *pfdn2*$^{\Delta 17}$ and *tub*-Gal4>UAS-Pfdn2 *pfdn2*$^{\Delta 17}$ were labeled with Dpn. (E) Larval brains of *pfdn2*$^{\Delta 10}$ and *insc*-Gal4>UAS-Pfdn2-Venus; *pfdn2*$^{\Delta 10}$ were labeled with Dpn. (F) Quantification of larval brain neuroblasts. *** indicates $p<0.001$. Error bars indicate mean standard deviation. NBs, neuroblasts. (G) Pfdn2 was labeled in wild-type and *pfdn2*$^{\Delta 10}$ larval brains. (H) Western blot of Pfdn2. Protein extracts from wild-type and *pfdn2*$^{\Delta 10}$ larval brains were probed by anti-Pfdn2. GAPDH is loading control. (I) Pfdn2 perdurance in *pfdn2*$^{\Delta 10}$ MARCM clones. Control and *pfdn2*$^{\Delta 10}$ MARCM clones were labeled with Pfdn2 and CD8. Clones are outlined by white dotted lines. (J) Larval brains of *insc*-Gal4>UAS-Pfdn2-Venus were labeled with Mira and DNA. The central brain (CB) is to the left of the white dotted line, which markers the border between the CB and the optic lobe. Scale bars: 20 μm (A, C-G), 5 μm (B, I-J).

Supplementary Figure 2

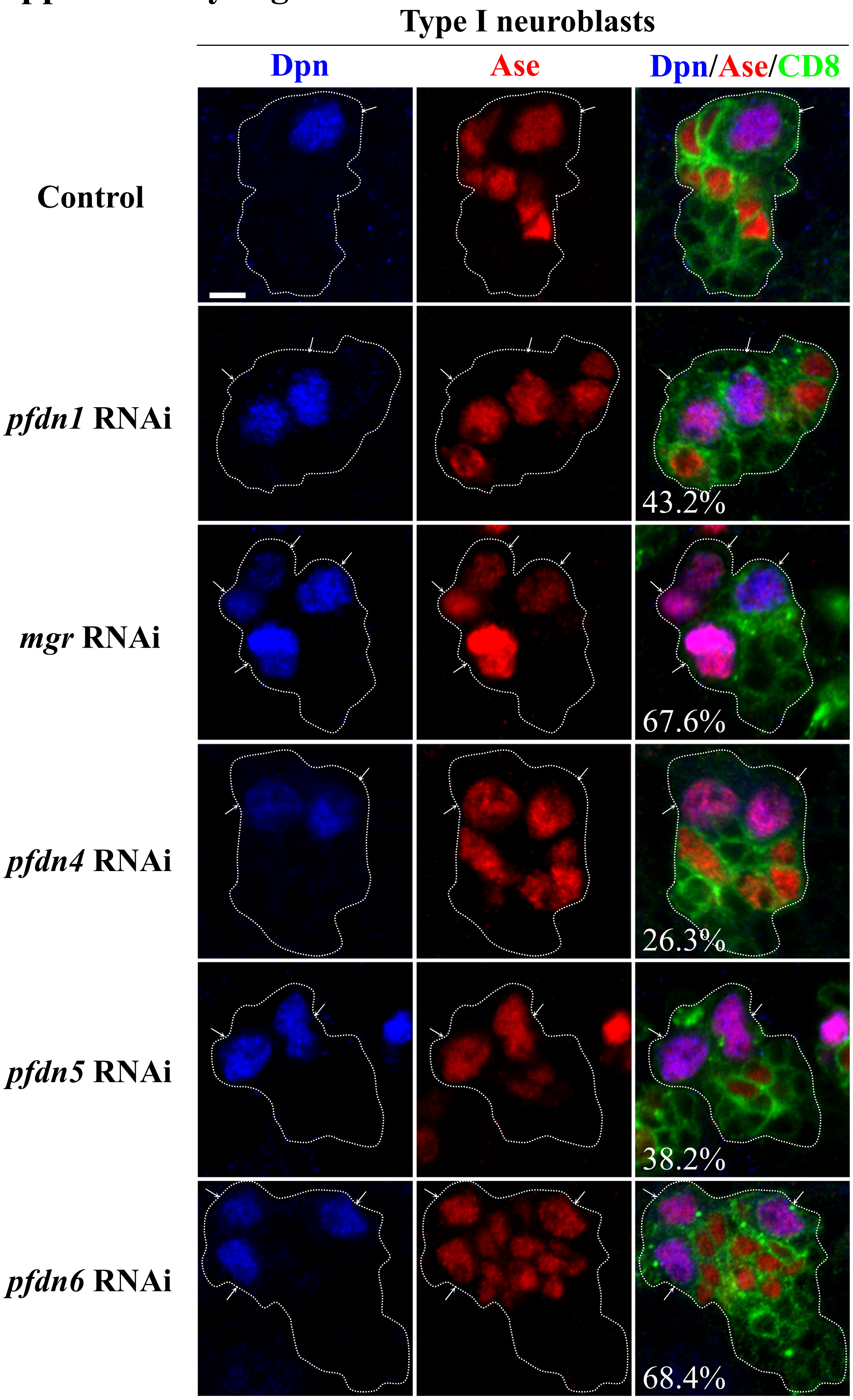

Supplementary Figure 2. Prefoldin complex is important for neuroblast self-renewal.
Driver control (*insc*-Gal4 UAS-CD8-GFP UAS-Dicer2), *pfdn1* RNAi, *mgr* RNAi, *pfdn4* RNAi, *pfdn5* RNAi and *pfdn6* RNAi were labeled with Dpn, Ase and CD8. Neuroblasts (Dpn^{+} Ase^{+} in type I lineages) in the clones are indicated by arrows. Clones are marked by CD8::GFP and outlined by white dotted lines. Scale bar: 5 μm.

Supplementary Figure 3

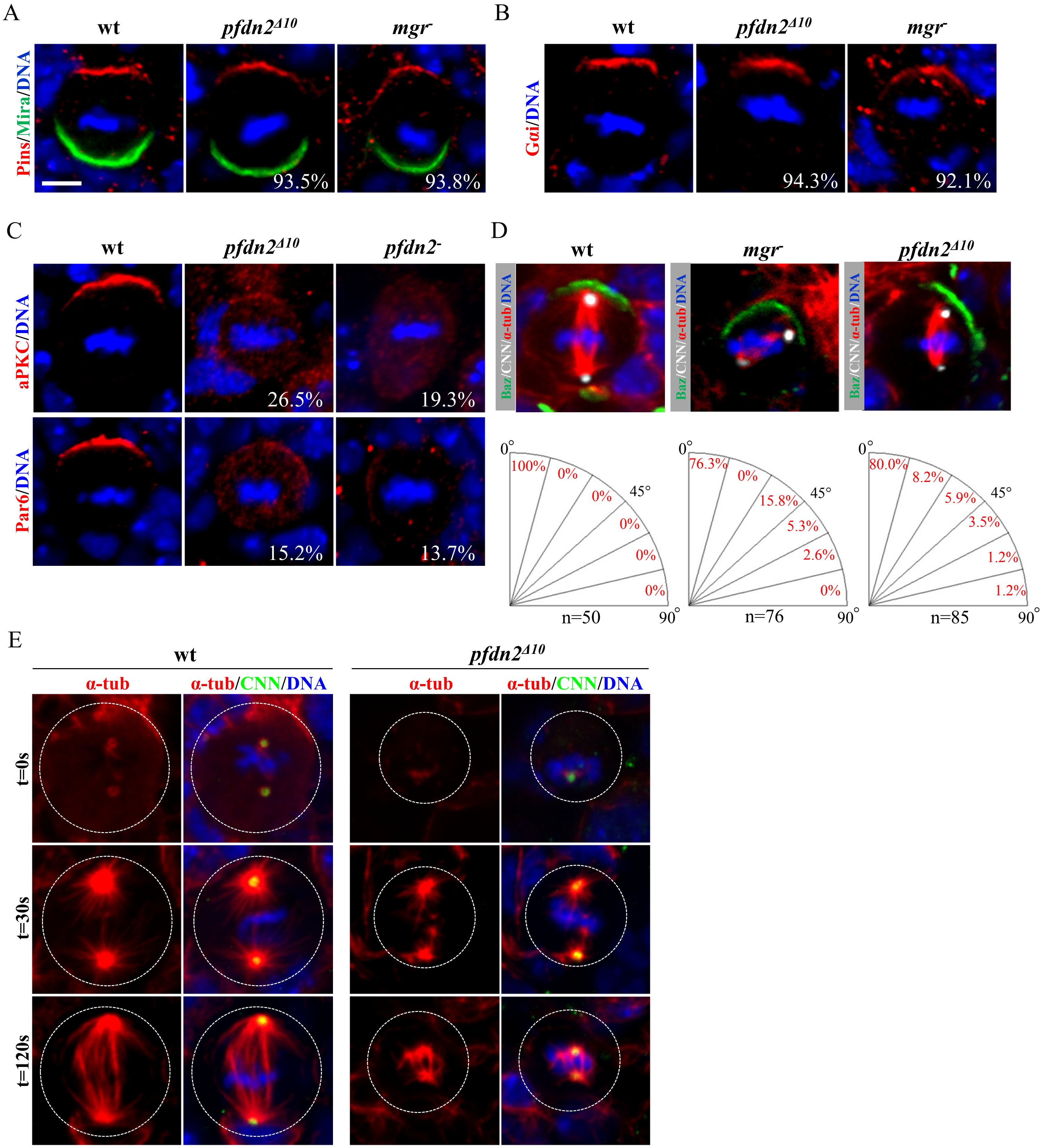

Supplementary Figure 3. Prefoldin regulates neuroblast asymmetric division and microtubule growth. (A) Wild-type control, *pfdn2*$^{\Delta 10}$ and *mgr*$^{-}$ [*mgr*^{G5308}*/Df(3R)Exel6160*] larval brains were labeled with Pins, Mira and DNA. (B) Wild-type control, *pfdn2*$^{\Delta 10}$ and *mgr*$^{-}$ [*mgr*^{G5308}*/Df(3R)Exel6160*] larval brains were labeled with Gαi and DNA. (C) aPKC, Par6 and DNA were labeled in wild-type, *pfdn2*$^{\Delta 10}$ and *pfdn2*$^{-}$ [*pfdn2*^{01239}*/Df(3L)BSC457*] larval brains. (D) Wild-type, *mgr*$^{-}$ [*mgr*^{G5308}*/Df(3R)Exel6160*] and *pfdn2*$^{\Delta 10}$ larval brain neuroblasts were labeled with Baz, CNN, α-tubulin and DNA. Mitotic spindle orientation was quantified. (E) Wild-type and *pfdn2*$^{\Delta 10}$ larval brain neuroblasts were labeled with CNN, α-tubulin and DNA. Neuroblasts were outlined by white dotted lines. Scale bar: 5 μm.

Supplementary Figure 4

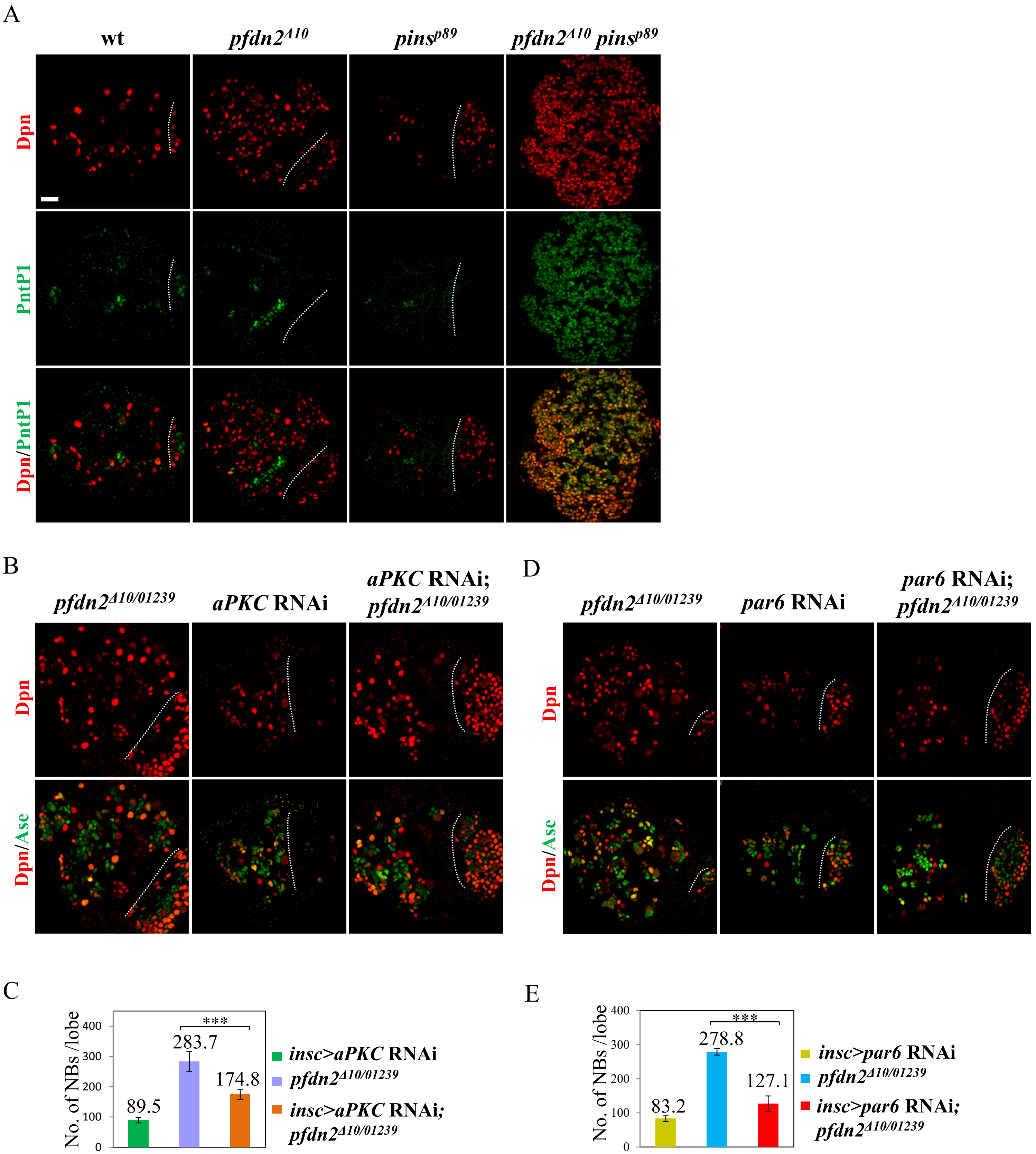

Supplementary Figure 4. Pfdn2 interacts with Pins to regulate neuroblast self-renewal.
(A) Dpn and PntP1 were labeled in wild-type control, *pins*^{p89}, *pfdn2*$^{\Delta 10}$ and *pfdn2*$^{\Delta 10}$ *pins*^{p89} larval brains. (B) *pfdn2*$^{\Delta 10/01239}$, *aPKC* RNAi under the *insc*-Gal4 UAS-Dicer2, and *aPKC* RNAi; *pfdn2*$^{\Delta 10/01239}$ under the *insc*-Gal4 UAS-Dicer2 larval brains were labeled with Dpn and Ase. (C) Quantification of larval brain neuroblasts. (D) *pfdn2*$^{\Delta 10/01239}$, *par6* RNAi under the *insc*-Gal4 UAS-Dicer2, and *par6* RNAi; *pfdn2*$^{\Delta 10/01239}$ under the *insc*-Gal4 UAS-Dicer2 larval brains were labeled with Dpn and Ase. (E) Quantification of larval brain neuroblasts. NBs, neuroblasts. The central brain (CB) is to the left of the white dotted line, which markers the border between the CB and the optic lobe. *** indicates $p<0.001$. Error bars indicate mean standard deviation. Scale bar: 20 μm

Supplementary Figure 5

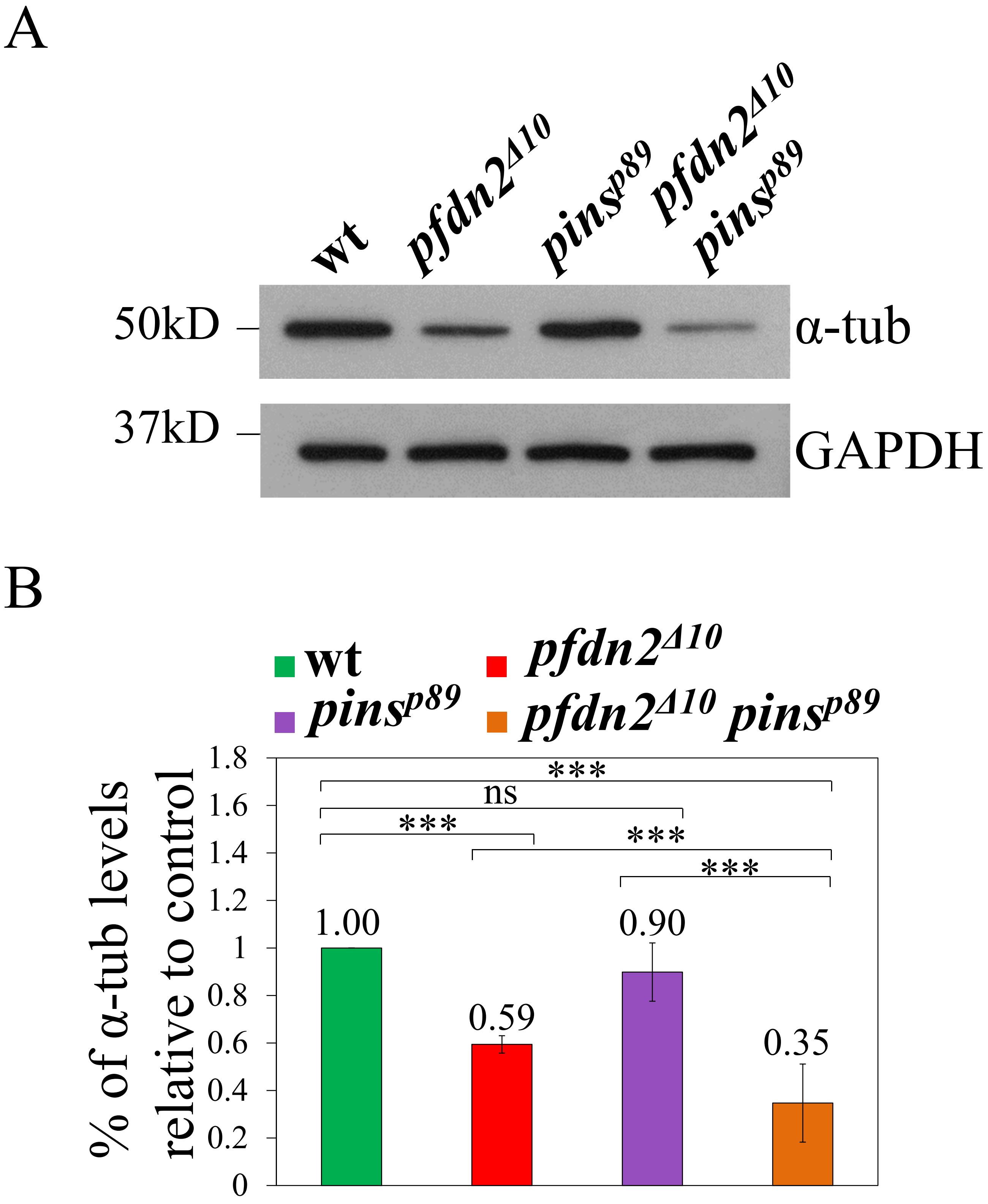

Supplementary Figure 5. Co-depletion of Pfdn2 and Pins results in further reduction of α-tubulin. (A) Western blot of α-tubulin. Protein extracts from wild-type control, *pfdn2*$^{\Delta 10}$, *pins*^{p89} and *pfdn2*$^{\Delta 10}$ *pins*^{p89} larval brains were probed by anti-α-tubulin. GAPDH is loading control. (B) Quantification of the expression level of α-tubulin. *** indicates $p<0.001$ (n=5). ns indicates $p>0.05$. Error bars indicate mean standard deviation.

Supplementary Figure 6

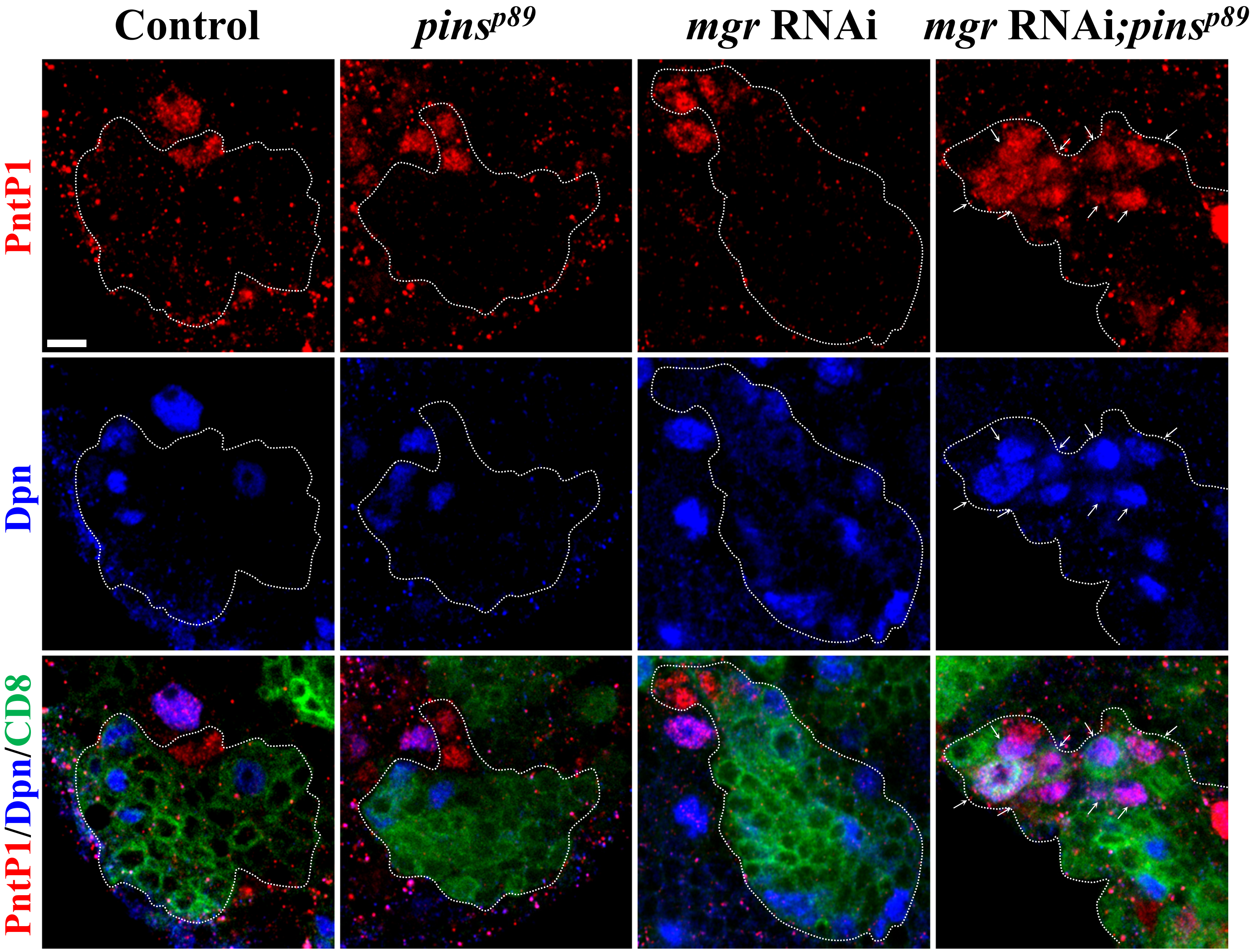

Supplementary Figure 6. Knockdown of *mgr* in *pins* mutant brains results in ectopic type II neuroblasts. Driver control (*erm*-Gal4 UAS-CD8-GFP), $pins^{p89}$, *mgr* RNAi UAS-Dicer2 and *mgr* RNAi UAS-Dicer2*;* $pins^{p89}$ larval brains were labeled with Dpn, PntP1 and CD8. Neuroblasts (Dpn^{+} $PntP1^{+}$ in type II lineages) in the clones are indicated by arrows. Clones are marked by CD8::GFP and outlined by white dotted lines. Scale bar: 5 μm.

Supplementary Figure 7

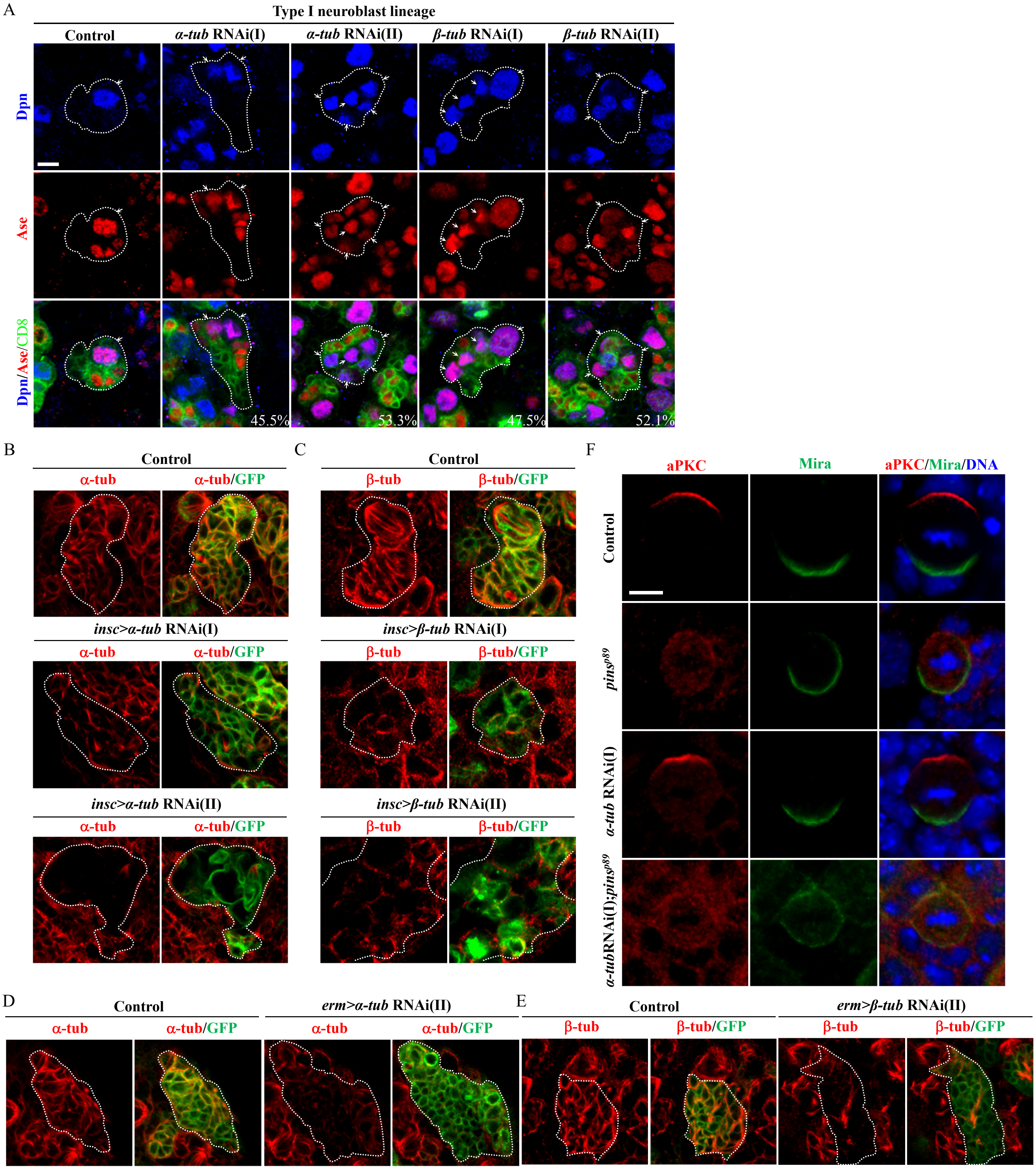

Supplementary Figure 7. Reduction of tubulin levels in *pins* mutant results in neuroblast overgrowth. (A) Driver control (*insc*-Gal4 UAS-CD8-GFP UAS-Dicer2), *α-tub* RNAi(I), *α-tub* RNAi(II), *β-tub* RNAi(I) and *β-tub* RNAi(II) were labeled with Dpn, Ase and CD8. Neuroblasts (Dpn^+ Ase^+ in type I lineages) in the clones are indicated by arrows. (B) Driver control (*insc*-Gal4 UAS-CD8-GFP), *α-tub* RNAi(I) and *α-tub* RNAi(II) were labeled with α-tubulin and CD8. (C) Driver control (*insc*-Gal4 UAS-CD8-GFP), *β-tub* RNAi(I) and *β-tub* RNAi(II) were labeled with β-tubulin and CD8. (D) Driver control (*erm*-Gal4 UAS-CD8-GFP) and *α-tub* RNAi(II) were labeled with α-tubulin and CD8. (E) Driver control (*erm*-Gal4 UAS-CD8-GFP) and *β-tub* RNAi(II) were labeled with β-tubulin and CD8. (F) Driver control (*insc*-Gal4), $pins^{p89}$, *α-tub* RNAi(I) and *α-tub* RNAi(I); $pins^{p89}$ larval brains were labeled with aPKC, Mira and DNA. Clones are marked by CD8::GFP and outlined by white dotted lines. Scale bar: 5 μm.

Supplementary Figure 8

A

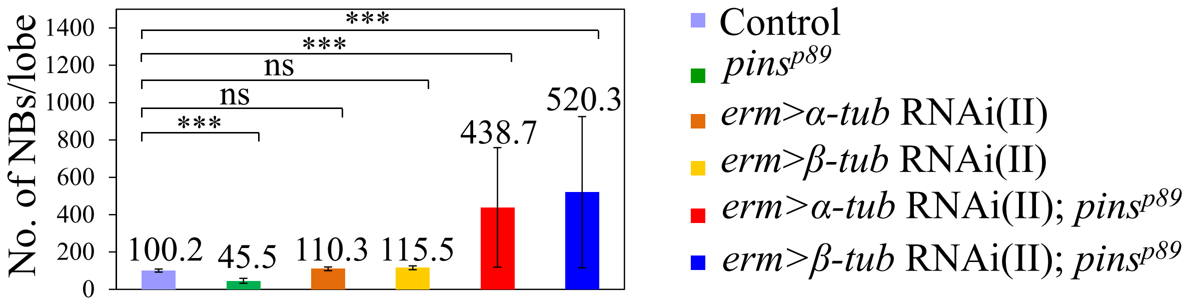

B

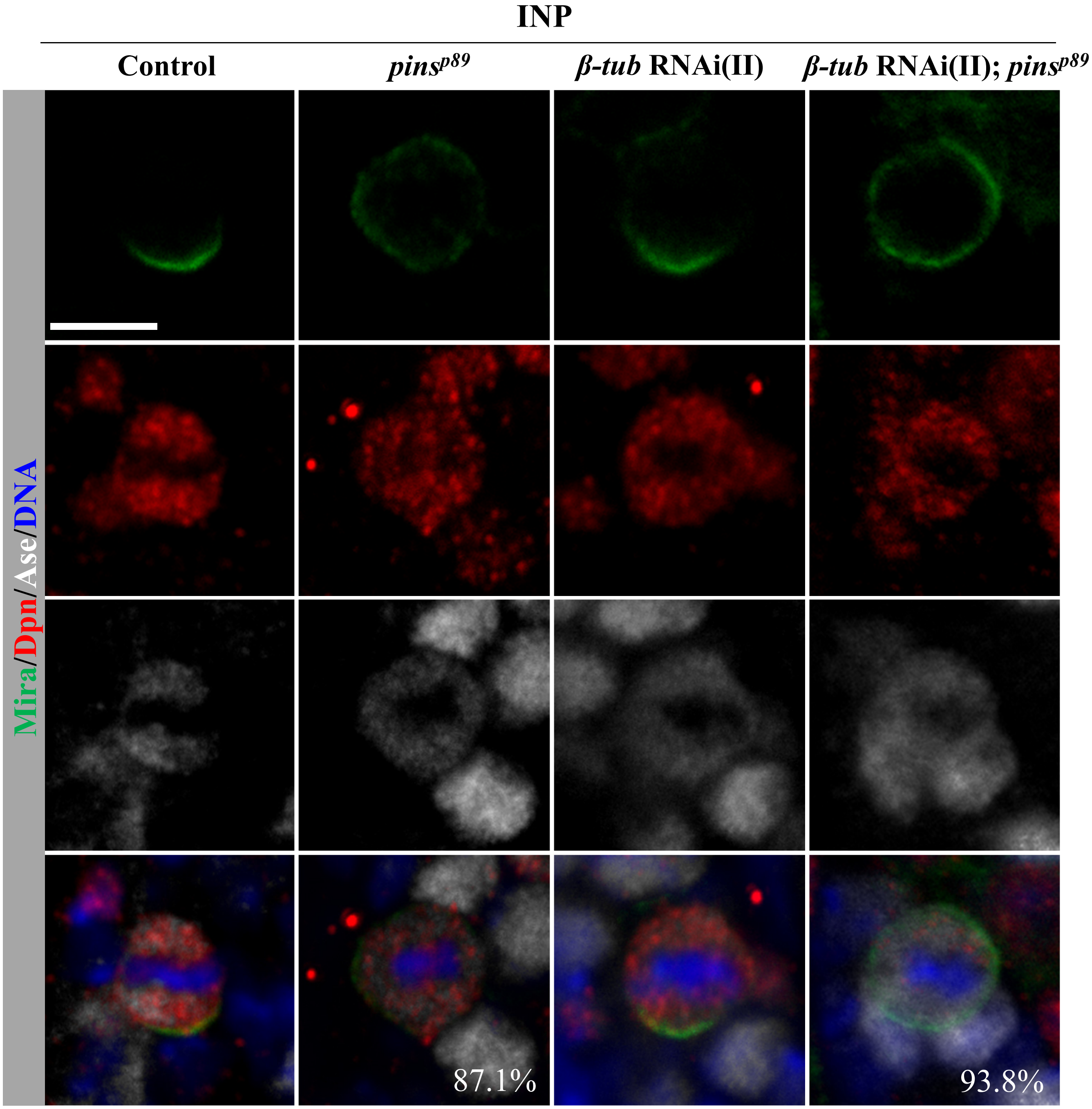

Supplementary Figure 8. Reduction of tubulin levels in *pins* mutant results in asymmetric division defects in INPs. (A) Quantification of neuroblast number. *** indicates p<0.001. ns indicates p>0.05. Error bars indicate mean standard deviation. NBs, neuroblasts. (B) Driver control (*erm*-Gal4 UAS-Dicer2), $pins^{p89}$, *β-tub* RNAi(II) and *β-tub* RNAi(II); $pins^{p89}$ larval brains were labeled with Dpn, Ase, Mira and DNA. Scale bar: 5 μm.

Supplementary Figure 9

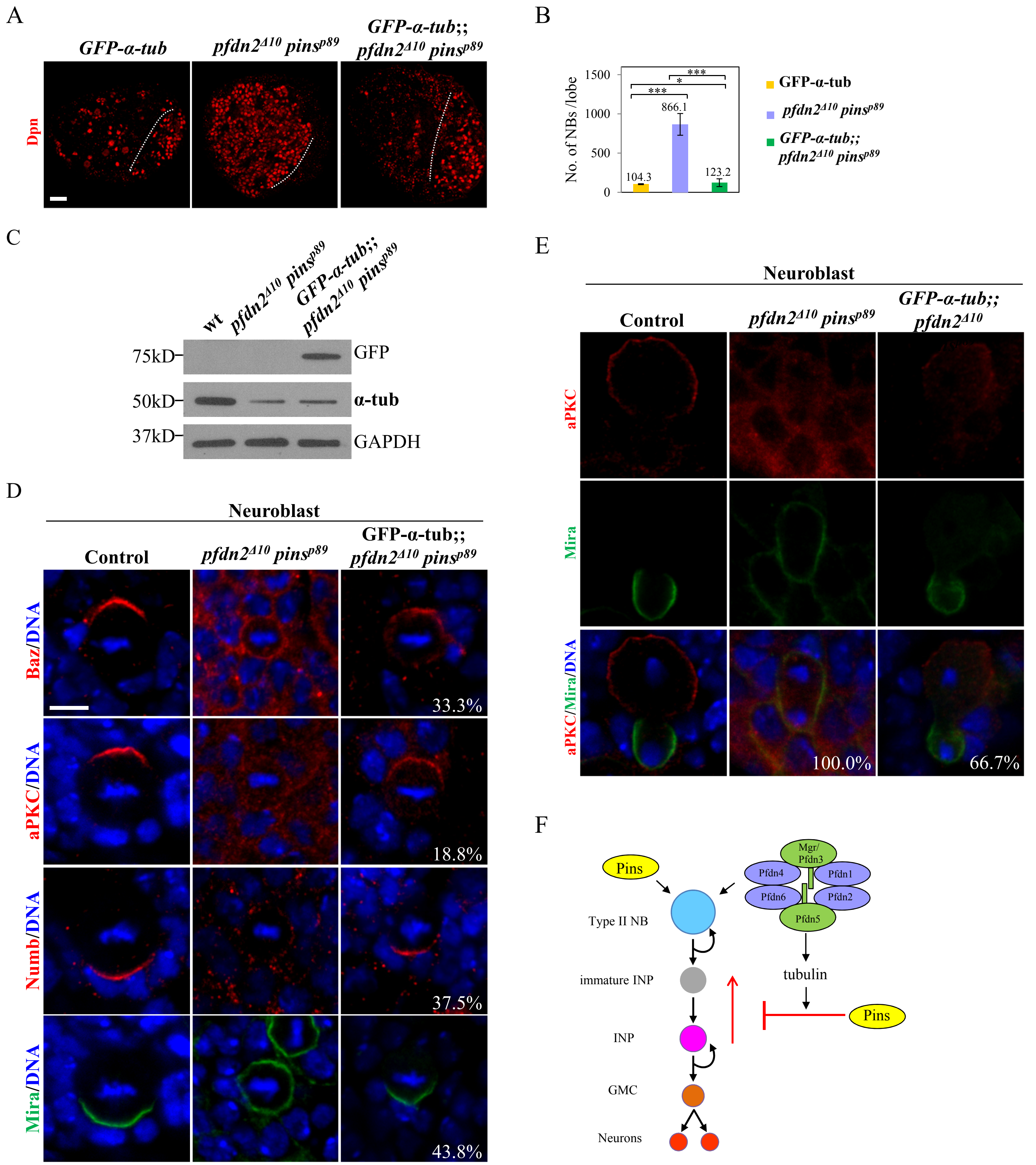

Supplementary Figure 9. Overexpression of α-tubulin suppresses neuroblast overgrowth and asymmetric division defects in *pfdn2 pins* double mutants. (A) Dpn was labeled in GFP-α-tub control, *pfdn2*$^{\Delta 10}$ *pins*^{p89} and GFP-α-tub;; *pfdn2*$^{\Delta 10}$ *pins*^{p89} larval brains. The central brain (CB) is to the left of the white dotted line, which markers the border between the CB and the optic lobe. (B) Quantification of larval brain neuroblasts. *** indicates $p<0.001$. Error bars indicate mean standard deviation. NBs, neuroblasts. (C) Western blot of α-tubulin. Protein extracts from wild-type, *pfdn2*$^{\Delta 10}$ *pins*^{p89}, and GFP-α-tub;; *pfdn2*$^{\Delta 10}$ *pins*^{p89} larval brains were probed by anti-α-tub and anti-GFP. GAPDH is loading control. (D) Baz, aPKC, Numb, Mira and DNA were labeled in wild-type control, *pfdn2*$^{\Delta 10}$ *pins*^{p89} and GFP-α-tub;; *pfdn2*$^{\Delta 10}$ *pins*^{p89} larval neuroblasts. (E) aPKC, Mira and DNA were labeled in wild-type control, *pfdn2*$^{\Delta 10}$ *pins*^{p89} and GFP-α-tub;; *pfdn2*$^{\Delta 10}$ *pins*^{p89} larval brains. (F) Model. Scale bars: 20 μm (A), 5 μm (D-E).

Supplementary Figure 10

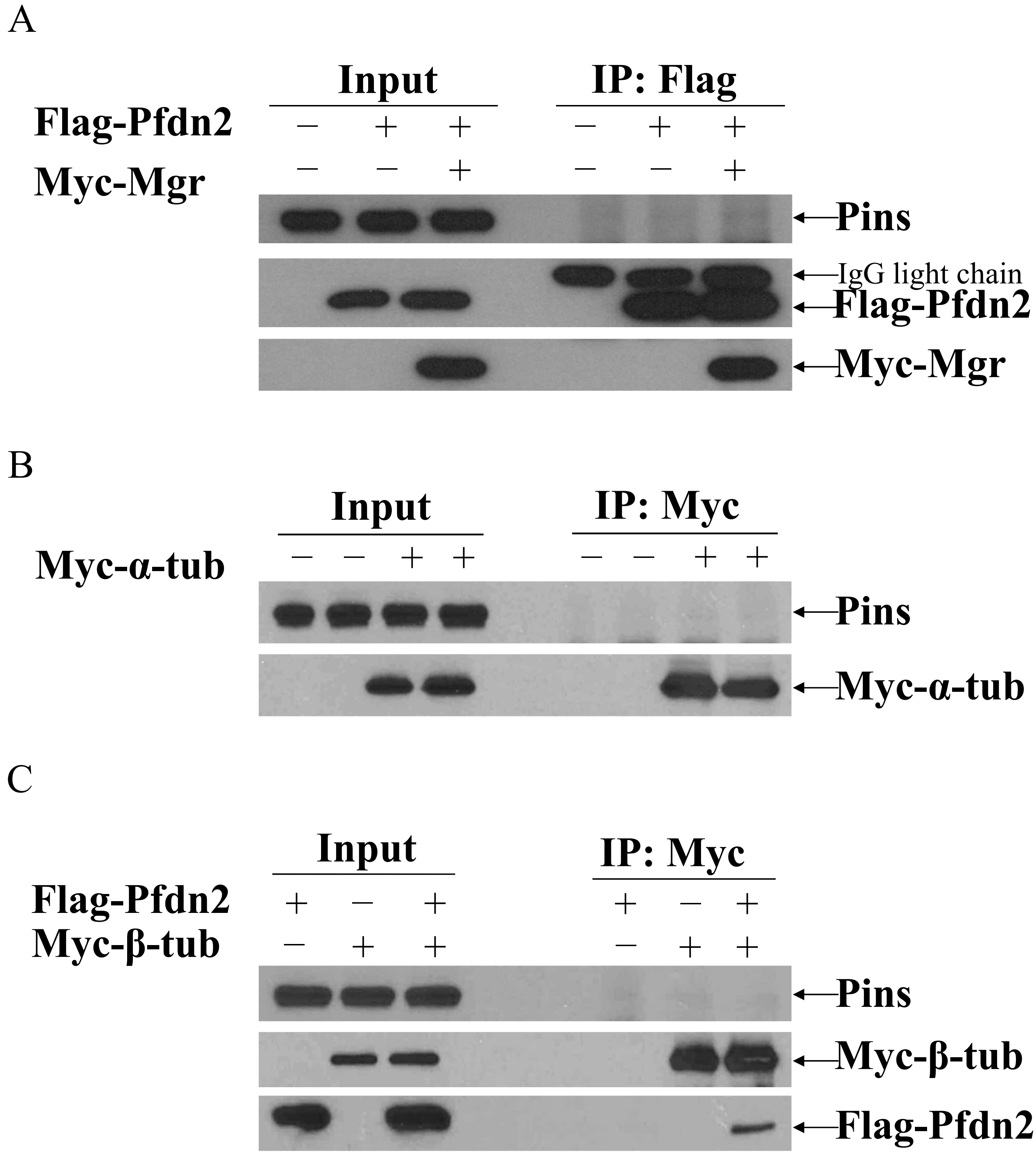

Supplementary Figure 10. Pins did not form a protein complex with Mgr, α-tubulin or β-tubulin in co-immunoprecipitation assays. Endogenous Pins was probed after immunoprecipitation of Flag or Myc-tagged proteins in S2 cells expressing (A) Flag-Pfdn2 and Myc-Mgr, (B) Myc-α-tubulin and (C) Flag-Pfdn2 and Myc-β-tubulin. Arrows indicate detected proteins or IgG light chain.
